# Blasticidin S Deaminase: A New Efficient Selectable Marker for *Chlamydomonas reinhardtii*

**DOI:** 10.3389/fpls.2020.00242

**Published:** 2020-03-05

**Authors:** Félix de Carpentier, Jeanne Le Peillet, Nicolas D. Boisset, Pierre Crozet, Stéphane D. Lemaire, Antoine Danon

**Affiliations:** ^1^Institut de Biologie Physico-Chimique, UMR 8226, CNRS, Sorbonne Université, Paris, France; ^2^Université Paris-Saclay, Saint-Aubin, France

**Keywords:** blasticidin, *Chlamydomonas reinhardtii*, antibiotic resistance, selectable marker, synthetic biology, modular cloning, algal biotechnology

## Abstract

*Chlamydomonas reinhardtii* is a model unicellular organism for basic or biotechnological research, such as the production of high-value molecules or biofuels thanks to its photosynthetic ability. To enable rapid construction and optimization of multiple designs and strains, our team and collaborators have developed a versatile Chlamydomonas Modular Cloning toolkit comprising 119 biobricks. Having the ability to use a wide range of selectable markers is an important benefit for forward and reverse genetics in Chlamydomonas. We report here the development of a new selectable marker based on the resistance to the antibiotic blasticidin S, using the *Bacillus cereus* blasticidin S deaminase (*BSR*) gene. The optimal concentration of blasticidin S for effective selection was determined in both liquid and solid media and tested for multiple laboratory strains. In addition, we have shown that our new selectable marker does not interfere with other common antibiotic resistances: zeocin, hygromycin, kanamycin, paromomycin, and spectinomycin. The blasticidin resistance biobrick has been added to the Chlamydomonas Modular Cloning toolkit and is now available to the entire scientific community.

## Introduction

*Chlamydomonas reinhardtii* is a model microalga widely used for basic and biotechnological research such as photosynthesis, cilia/flagella, production of biofuels, or other molecules of interest (Georgianna and Mayfield, 2012; Barahimipour et al., 2016; Salomé and Merchant, 2019). In the last decades Chlamydomonas has been shown to be amenable to powerful genetic approaches including CRISPR-Cas9 gene editing (Jiang et al., 2014; Greiner et al., 2017; Kao and Ng, 2017). The creation of the Chlamydomonas Library Project (CLiP), which contains more than 62,000 mutants covering roughly 83% of Chlamydomonas genes (Li et al., 2019), has also greatly contributed to the development of Chlamydomonas reverse genetics. A wide range of molecular tools for engineering of the nuclear genome are also available in Chlamydomonas, most of which have been grouped in a Modular Cloning toolkit (Chlamy MoClo toolkit). This collection contains 119 biobricks (promoters, terminators, reporter genes, selectable markers, targeting peptides, antibiotic resistance genes, riboswitch, miRNA backbone, etc.), which can be easily assembled through Golden Gate cloning (Crozet et al., 2018). Most applications related to the manipulation of nuclear gene expression require transformation of Chlamydomonas cells followed by selection of transformants on plates using a selectable marker, which is in most cases an antibiotic resistance gene enabling selection on a medium containing the appropriate antibiotic. In the case of multiple consecutive transformations several selectable markers are required. Therefore, it is essential for advanced genetic engineering to have as many selectable markers as possible. At the moment, six antibiotic resistances are commonly used in Chlamydomonas as selectable markers: zeocin (Stevens et al., 1996), hygromycin (Berthold et al., 2002), kanamycin (Barahimipour et al., 2016), paromomycin (Sizova et al., 2001), sulfadiazine (Tabatabaei et al., 2019), and spectinomycin (Meslet-Cladière and Vallon, 2011).

Blasticidin S (hereafter referred to as “blasticidin”) is an antibiotic that inhibits cytosolic protein synthesis by blocking ribosomal translation termination (Svidritskiy et al., 2013). Blasticidin S deaminase (BSR) detoxifies blasticidin by catalyzing its deamination (Seto et al., 1966; Endo et al., 1987). The *BSR* gene has been successfully used as a selectable marker in mammals (Izumi et al., 1991), plants (Kamakura et al., 1990), yeasts (Kimura et al., 1994; Fukuda and Kizaki, 1999), and algae, including the diatom *Phaeodactylum tricornutum* (Buck et al., 2018) and the Chlamydomonas-related volvocine alga *Volvox carteri* (Ortega-Escalante et al., 2018).

We report here that blasticidin can be used as an antibiotic in Chlamydomonas, for all six laboratory strains tested. We show that BSR is functional in Chlamydomonas and can be used as a selectable marker, without conferring resistance to the other commonly used antibiotics. We also show that BSR can be used in combination with all other antibiotic resistance genes available in Chlamydomonas.

## Materials and Methods

### Strains, Media, and Growth Conditions

If not otherwise specified, the reference strain used in the present study is CC-4533 (CMJ030), the CLiP library recipient strain used to generate the insertional mutants (Li et al., 2019). We also used other common laboratory strains CC-4051 (4A+) (Kim et al., 2005), CC-400 (cw15) (Roy Davies, 1972), CC-4425 (D66) (Schnell and Lefebvre, 1993), UVM4 (Neupert et al., 2009), and CC-124 (137c) (Pröschold et al., 2005). Chlamydomonas cells were grown on agar plates or liquid medium, using Tris-acetate-phosphate (TAP) medium (Gorman and Levine, 1965) at 25°C, under continuous light (40–60 μmol photon⋅m^–2^⋅s^–1^) and shaking (130 rpm). Growth analysis were performed in the Algem^®^ labscale double photobioreactor systems (Algenuity, Stewartby, United Kingdom) under continuous light (200 μmol photons⋅m^–2^⋅s^–1^) and 120 rpm agitation in TAP or high salt medium (HSM) (Sueoka, 1960), with bubbling air. The absorbance at 740 nm was recorded every 10 min using the built-in sensor. The maximal growth rate was determined as the maximal slope of the growth curve (Δabs/Δtime). Linearity of the growth curve was estimated through linear regression every 200 min over a period of 13 h for TAP or 300 min over a period of 40 h for HSM condition. The slopes of these linear regressions were selected only for regressions displaying an *R*^2^ < 99.5%. Finally, the maximal slope was chosen as the maximal growth rate (Supplementary Figure S2B). Antibiotics used were blasticidin S (Sigma-Aldrich: SBR00022), zeocin (Invitrogen: R25005), hygromycin B (Sigma-Aldrich: H9773), kanamycin (Sigma-Aldrich: K1377), paromomycin (Sigma-Aldrich: P8692), or spectinomycin (Sigma-Aldrich: S4014). Chlamydomonas multi-well plates and Petri dishes were scanned using a Perfection V800 scanner (Epson).

### Cell Death Quantification

Dead cells were detected using Evans blue (Sigma-Aldrich: E2129) at a final concentration of 0.2% w/v. Dead cells appear in blue whereas living cells that are impermeable to Evans blue remain green (Gaff and Okong’o-Ogola, 1971). Cells were observed with a microscope (Olympus BX43, Tokyo, Japan). For each sample multiple microscopic fields were analyzed and Evans blue-positive cells scored. For each value the percentage of dead cells was calculated on a minimum of 100 individuals.

### Plasmid Construction

Protein and nucleic acid designs were performed *in silico* on Serial Cloner 2.6.1 software. *Bacillus cereus* BSR protein sequence (NCBI accession number: WP_076871832.1) was reverse translated using Chlamydomonas nuclear genome codon usage table^1^. The resulting *BSR* coding sequence was domesticated by removing *Bbs*I restriction sites, designed for the position B3–B5 of the common Plant MoClo syntax (Patron et al., 2015), synthetized (Twist Bioscience), and cloned by Golden Gate reaction with *Bsa*I-HFv2 (New England Biolabs) in pICH41308 (Weber et al., 2011) yielding plasmid pCM0-120, numbered according to the Chlamydomonas MoClo toolkit nomenclature (Crozet et al., 2018). Two other parts from the toolkit were used to build the *BSR* module (pCM1-029), the promoter P_A/R_ coupled to the 5′UTR of RBCS2 (pCM0-020) and the 3′UTR of RBCS2 coupled to terminator T_RBCS__2_ (pCM0-115) (Schroda et al., 2002; Crozet et al., 2018). The other antibiotic resistance genes were built using these same regulatory sequences and the coding sequence (CDS) from pCM0-077 (zeocin), pCM0-073 (hygromycin), pCM0-074 (paromomycin), pCM0-075 (kanamycin) and pCM0-076 (spectinomycin) generating the plasmids pCM1-030 (zeocin), pCM1-031 (hygromycin), pCM1-032 (kanamycin), pCM1-027 (paromomycin), and pCM1-033 (spectinomycin), respectively. All plasmid sequences are available in Supplementary Files S1–S7.

### Chlamydomonas Transformation

Transformations were performed as previously described (Crozet et al., 2018). Briefly, Chlamydomonas cells were grown in TAP to early exponential phase (1–2 × 10^6^ cells/mL), concentrated 100 times in TAP + 60 mM sucrose. After incubation of 250 μL of cells with DNA (55 fmol of purified resistance module excised with *Bbs*I-HF; New England Biolabs) at 4°C for 20 min, they were electroporated (2000 V/cm, 25 μF, no shunt resistance) and incubated for 16–20 h in 10 mL of TAP + 60 mM sucrose prior to be plated on TAP-agar complemented with appropriate antibiotic(s). Transformants were selected on TAP-agar medium containing blasticidin S (50 mg/L, unless otherwise specified), zeocin (15 mg/L), hygromycin B (20 mg/L), kanamycin (50 mg/L), paromomycin (20 mg/L), and/or spectinomycin (100 mg/L). Plates and transformants were analyzed after 5–7 days of growth in continuous light (50 μmol photon m^–2^ s^–1^) at 25°C.

### Insert Detection

Cells were pelleted (5 min, 2500 × *g*, room temperature) and lysed in 400 μL of extraction buffer (200 mM Tris-HCl pH 7.5; 200 mM NaCl; 25 mM EDTA; 0.5% SDS) for 10 min at 37°C under agitation (1400 rpm). After centrifugation (3 min, 17,000 × *g*, room temperature), the supernatant was harvested and the genomic DNA was precipitated with one volume of isopropanol for 10 min at room temperature, washed with 70% ethanol, dried and resuspended in water. PCR was performed using the Quick-Load^®^ *Taq* 2× Master Mix (New England Biolabs) according to the manufacturer recommendations with the primers BSR.5 (5′-GCTGTACGAGGACAACAAGC-3′), TRBCS2.3 (5′-ACGGAGGATCGTTACAACC-3′), CBLP.5 (5′-GACGTCATCCACTGCCTGTG-3′), and CBLP.3 (5′-CGACGCATCCTCAACACACC-3′).

## Results

### Chlamydomonas Is Sensitive to Blasticidin

**FIGURE 1:**
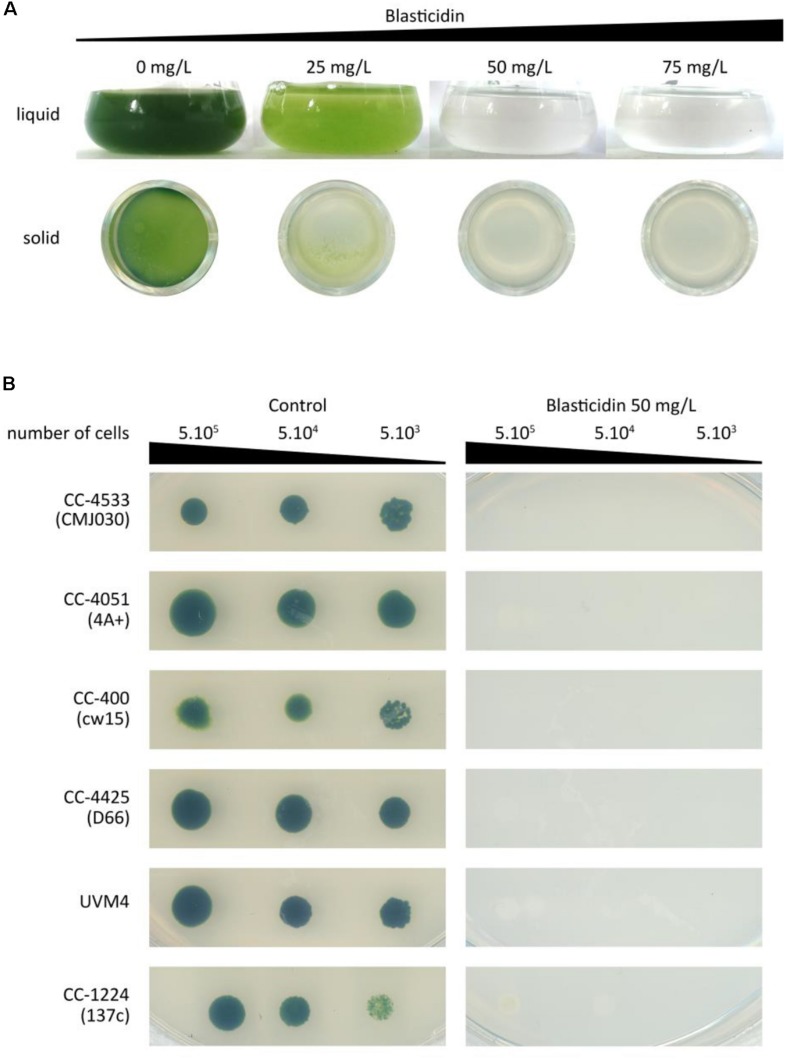
Sensitivity of Chlamydomonas to blasticidin. **(A)** Chlamydomonas CC-4533 (CMJ030) was grown with increasing concentrations of blasticidin: 25, 50, or 75 mg/L, in both liquid and 24-well agar plates. For both liquid and solid media, cultures were photographed after 3 days of growth. **(B)** Spot tests were performed for six standard laboratory strains with or without blasticidin (50 mg/L). The results shown are representative of three biological replicates.

To test the sensitivity of Chlamydomonas to blasticidin, the cells were grown in the presence of increasing blasticidin concentrations (25, 50, or 75 mg/L) in both solid and liquid cultures. Blasticidin was very effective to kill Chlamydomonas cells and the minimum efficient concentration was 50 mg/L in both conditions (Figure 1A). Many reference strains are used within the community and they present an important genetic diversity and substantial phenotypic differences (Gallaher et al., 2015). Compared to the reference strain of this study (CC-4533), some strain specific phenotypes, such as the absence of the cell wall, could alter blasticidin resistance. To assess whether this genetic diversity among common reference strains affects their sensitivity to blasticidin, CC-4051 (4A+), CC-400 (cw15), CC-4425 (D66+), UVM4, and CC-124 (137c) were cultivated on a solid medium supplemented or not with blasticidin (50 mg/L). In all cases, none of the strains survived in the presence of blasticidin, regardless of the initial number of cells tested (Figure 1B).

### Blasticidin S Deaminase Can Be Used as a Selectable Marker in Chlamydomonas

**FIGURE 2:**
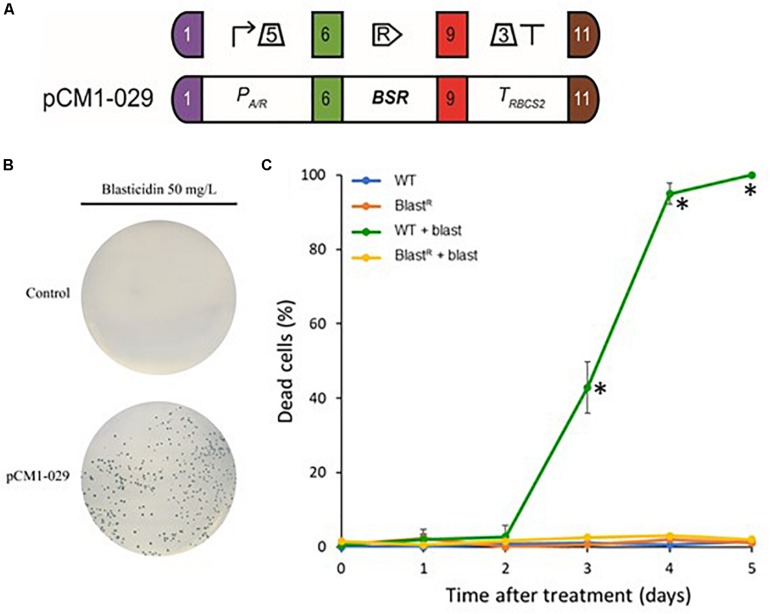
Blasticidin S deaminase expression in Chlamydomonas. **(A)** The blasticidin resistance module pCM1-029 is built from a constitutive promoter (P_A/R_ + 5′UTR of RBCS2), the BSR coding sequence and a terminator (3′UTR of RBCS2 + T_RBCS__2_) and was assembled using Golden Gate cloning. The numbers 1, 6, 9, and 11 stand for the standard fusion sites used for cloning (Crozet et al., 2018). SBOL2.0 visual syntax is shown above the module (Roehner et al., 2016). **(B)** CC-4533 cells were transformed with pCM1-029 or an empty vector (pICH47742) (Weber et al., 2011) and plated on TAP-agar supplemented with blasticidin (50 mg/L). Results are representative of three biological replicates. **(C)** The percentage of dead cells was evaluated using Evans blue in wild-type and Blast^R^ cultures with or without blasticidin, several days after treatment. Values represent the means and standard deviations of three independent experiments, symbol (^∗^) shows the samples found to be significantly different doing an ANOVA analysis for each time points (*p* < 0.001).

To test whether the *BSR* module could be used as a selectable marker in Chlamydomonas, we engineered *B. cereus BSR* coding sequence to fit the optimal codon bias of Chlamydomonas nuclear genome. This has been already shown to improve transgene expression efficiency, including selection markers (Barahimipour et al., 2015, 2016). The engineered *BSR* coding sequence was then domesticated by removing *Bbs*I and *Bsa*I recognition motif and adding appropriate fusion sites of the Plant MoClo syntax (Patron et al., 2015). It was finally assembled with the parts P_A/R_ (the hybrid promoter of *HSP70A* coupled to the 5′UTR of *RBCS2*) and T_RBCS__2_ (coupling the 3′UTR and terminator of *RBCS2*) to form a functional module. P_A/R_ is a chimeric constitutive promoter made up of *HSP70A* and *RBCS2* promoters that was proven to be very efficient in Chlamydomonas by significantly reducing gene silencing (Schroda et al., 2002). We also chose this promoter/terminator combination because it allows successful expression of the same *BSR* gene in Volvox (Ortega-Escalante et al., 2018). The resulting construct pCM1-029 (pCM stands for plasmid Chlamydomonas MoClo) is represented in Figure 2A using the MoClo nomenclature (Crozet et al., 2018). pCM0-120 and pCM1-029 plasmid sequences are available in Supplementary Files S1, S2, respectively. Cells transformed with pCM1-029 or an empty vector were incubated on plates containing blasticidin. Transformants appeared only when the cells were transformed with pCM1-029 (Blasticidin resistant cells are hereafter referred to as “Blast^R^”), as shown in Figure 2B, indicating that *BSR* can be used as a selectable marker in Chlamydomonas. The insertion of the *BSR* module was confirmed by PCR in four independent Blast^R^ strains (Supplementary Figure S1).

To precisely evaluate the effect of blasticidin on the mortality of wild-type and Blast^R^ cells, we used Evans blue as a death marker (Gaff and Okong’o-Ogola, 1971). To avoid the heterogeneity that comes from the position of the transgene insertion locus, dozens of Blast^R^ colonies were collected from a Petri dish and then mixed in liquid culture (Blast^R^ culture). Wild-type and Blast^R^ cultures were grown in a 24-well plate with or without blasticidin, and the percentage of dead cells was evaluated several days after treatment. In the wild-type strain treated with blasticidin, the percentage of dead cells increased after 3 days and all the cells died after 5 days, while no significant levels of death was detected for Blast^R^ and untreated cells (Figure 2C). To assess a potential detrimental effect of BSR on Chlamydomonas, wild-type and Blast^R^ growth were quantified in a photobioreactor, in both mixotrophic (TAP) and autotrophic (HSM) conditions. No differences in the growth rate were observed suggesting that BSR does not affect growth or photosynthesis (Supplementary Figure S2).

### Blasticidin Can Be Used in Combination With Other Antibiotics

**FIGURE 3:**
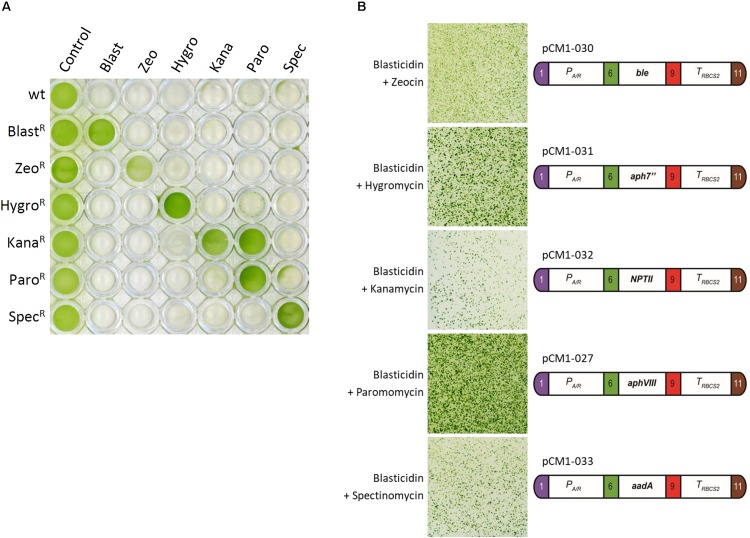
Cross reactivity test. **(A)** Liquid cultures in exponential phase of wild-type strain and Zeo^R^, Hygro^R^, Kana^R^, Paro^R^, and Spec^R^ cultures were placed in a 96-well plate and treated with blasticidin (Blast, 50 mg/L), zeocin (Zeo, 15 mg/L), hygromycin B (Hygro, 20 mg/L), kanamycin (Kana, 50 mg/L), paromomycin (Paro, 20 mg/L), and spectinomycin (Spec, 100 mg/L) for 5 days. **(B)** A blasticidin resistant strain carrying pCM1-029 was independently transformed with the resistance modules to zeocin (pCM1-030), hygromycin (pCM1-031), kanamycin (pCM1-032), paromomycin (pCM1-027), and spectinomycin (pCM1-033). The numbers 1, 6, 9, and 11 stand for the standard fusion sites used for cloning (Crozet et al., 2018). Transformants were selected on plate containing both blasticidin and the appropriate antibiotic. The results showed are representative of three biological replicates. The full experiment with the different controls is available in Supplementary Figure S3.

To test the possible interactions of blasticidin with the other most commonly used selectable markers in Chlamydomonas, we first generated strains resistant to these antibiotics by transformation of wild-type cells with plasmids containing modules conferring resistance to zeocin (pCM1-030), hygromycin (pCM1-031), kanamycin (pCM1-032), paromomycin (pCM1-027), and spectinomycin (pCM1-033) under the control of the same regulatory sequences used for *BSR* in pCM1-029. Transformants were selected on plates containing the appropriate antibiotic and pooled to take into account position effect, as for Blast^R^ culture. These cultures were called Zeo^R^, Hygro^R^, Kana^R^, Paro^R^, and Spec^R^. The wild-type strain and the six antibiotic resistant cultures were cultivated in 96-well plates until exponential phase (5 × 10^6^ cells/mL) prior to treatment with the different antibiotics. Five days after the addition of antibiotics, only the cells carrying the corresponding resistance gene survived the treatment, and importantly, only the Blast^R^ culture had survived upon blasticidin treatment (Figure 3A). It is to be noticed that Kana^R^ strains are also resistant to paromomycin (Figure 3A), as previously reported (Barahimipour et al., 2016).

If the resistance modules tested display no cross reactivity to one another, they can be combined to allow double selection. To use the *BSR* module and blasticidin in combination with another selectable marker, there must then be no interference between the different antibiotics and resistance modules. To verify possible interference, a single Blast^R^ strain was transformed with either pCM1-030 (zeocin resistance), pCM1-031 (hygromycin resistance), pCM1-032 (kanamycin resistance), pCM1-027 (paromomycin resistance), or pCM1-033 (spectinomycin resistance) (Figure 3B). Transformants were selected on plates containing both blasticidin and the appropriate antibiotic. For each combination tested, transformants resistant to both antibiotics could be obtained (Figure 3B), indicating that no interference exists between the selectable markers tested.

## Discussion

Here we report the development of a new selectable marker for *C. reinhardtii*. Our data show that using blasticidin at a concentration of 50 mg/L ensures proper selection for all common laboratory strains of Chlamydomonas (Figure 2C). This concentration is slightly higher than the efficient concentration reported for *V. carteri* (Ortega-Escalante et al., 2018) or diatoms (Buck et al., 2018), but remains comparable with other antibiotics used in Chlamydomonas (Crozet et al., 2018).

We successfully engineered *BSR* coding sequence to adapt it to Chlamydomonas and no addition of intron was necessary for efficient expression, contrary to what was reported for *V. carteri* (Ortega-Escalante et al., 2018). Now that we have developed the blasticidin resistance module, seven antibiotic-based selectable markers are available for Chlamydomonas. This new tool can become important for advanced synthetic biology strategies requiring successive transformations of the same strain in combination with new engineering tools including the CLiP library (Li et al., 2019), the CRISPR/Cas technology (Jiang et al., 2014; Greiner et al., 2017; Kao and Ng, 2017) and the MoClo toolkit (Crozet et al., 2018). It is also important to increase the number of selectable markers available because the use of certain antibiotics should be taken with care. For instance zeocin is not always recommended since it can potentially cause DNA damages (Chankova et al., 2007; Čížková et al., 2019) and subsequent unwanted mutations. The kanamycin selectable marker *NPTII* is also conferring resistance to paromomycin (Barahimipour et al., 2015; Figure 3A) which makes it impossible to use in CLiP strains that are paromomycin resistant (Li et al., 2019). We show here that it is possible to use *BSR* and blasticidin in combination with all the other commonly used selectable markers in Chlamydomonas. *BSR* gene has been integrated as a new biobrick into the Chlamydomonas MoClo toolkit, and is now available to the entire community through the Chlamydomonas Resource Center^2^.

## Data Availability Statement

All datasets generated for this study are included in the article/Supplementary Material.

## Author Contributions

FC, JL, PC, SL, and AD designed the study and analyzed the data. FC, JL, NB, PC, SL, and AD wrote the manuscript. FC, JL, NB, and AD performed the experiments.

## Conflict of Interest

The authors declare that the research was conducted in the absence of any commercial or financial relationships that could be construed as a potential conflict of interest.

## Footnotes

1 www.kazusa.or.jp/codon/cgi-bin/showcodon.cgi?species=3055

2 www.chlamycollection.org/

## Supplementary Material

FIGURE S1 Detection of the *BSR* module. pCM1-029 plasmid and the genomic DNA of the wild type (CC-4533) and four independent transformants Blast^R^ were used to amplify by PCR a fragment of the *BSR* resistance module. A product at the expected size (546 bp) was amplified only for pCM1-029 (positive control) and the Blast^R^ transformants. Amplification of CBLP (Cre06.g278222) was used as a positive control for the experiment.

FIGURE S2 Growth of wild-type (CC-4533) and Blast^R^ strains in a controlled photobioreactor. **(A)** Measurement of growth as detected by absorbance at 740 nm every 10 min, in TAP (mixotrophy) and HSM medium (autotrophy), light was set to 200 μmol photons⋅m^–2^⋅s^–1^ with temperature and shaking set at 25°C and 120 rpm, respectively. **(B)** Quantification of the average growth rate of wild-type and Blast^R^ cells, corresponding to the growth curves shown in Graph **(A)**. Error bars represent the standard error to the mean of two biological replicates.

FIGURE S3 Transformation of a blasticidin resistant strain with zeocin, hygromycin, kanamycin, paromomycin and spectinomycin resistance plasmids. A blasticidin resistant strain (Blast^R^) carrying pCM1-029 was independently transformed with the resistance modules to zeocin (pCM1-030), hygromycin (pCM1-031), kanamycin (pCM1-032), paromomycin (pCM1-027), and spectinomycin (pCM1-033) or an empty vector (pICH50881). The numbers 1, 6, 9, and 11 stand for the standard fusion sites used for cloning (Crozet et al., 2018). Blast^R^ transformed cells were selected on plate containing both blasticidin and the appropriate antibiotic. CC-4533 was also transformed with the same five resistance modules or the empty vector and selected on the corresponding antibiotic with or without addition of blasticidin. The results showed are representative of three biological replicates.

FILE S1 Annotated sequence of pCM0-120, the level 0 plasmid containing *BSR* coding sequence engineered for Chlamydomonas nuclear genome and designed for the position B3–B5 of the Chlamydomonas MoClo toolkit (Crozet et al., 2018).

FILE S2 Annotated sequence of pCM1-029, the level 1 plasmid made up of the promoter AR (P_A/R_ + 5′UTR of RBCS2), *BSR* coding sequence and the terminator of RBCS2 (3′UTR of RBCS2 + T_RBCS2_) conferring blasticidin resistance in Chlamydomonas (Crozet et al., 2018).

FILE S3 Annotated sequence of pCM1-027, the level 1 plasmid made up of the promoter AR (P_A/R_ + 5′UTR of RBCS2), *aphVIII* coding sequence and the terminator of RBCS2 (3′UTR of RBCS2 + T_RBCS2_) conferring paromomycin resistance in Chlamydomonas (Sizova et al., 2001; Crozet et al., 2018).

FILE S4 Annotated sequence of pCM1-030, the level 1 plasmid made up of the promoter AR (P_A/R_ + 5′UTR of RBCS2), *Ble* coding sequence and the terminator RBCS2 (3′UTR of RBCS2 + T_RBCS2_) conferring zeocin resistance in Chlamydomonas (Stevens et al., 1996; Crozet et al., 2018).

FILE S5 Annotated sequence of pCM1-031, the level 1 plasmid made up of the promoter AR (P_A/R_ + 5′UTR of RBCS2), *aph7*″ coding sequence and the terminator RBCS2 (3′UTR of RBCS2 + T_RBCS2_) conferring hygromycin resistance in Chlamydomonas (Berthold et al., 2002; Crozet et al., 2018).

FILE S6 Annotated sequence of pCM1-032, the level 1 plasmid made up of the promoter AR (P_A/R_ + 5′UTR of RBCS2), *NPTII* coding sequence and the terminator RBCS2 (3′UTR of RBCS2 + T_RBCS2_) conferring kanamycin resistance in Chlamydomonas (Barahimipour et al., 2016; Crozet et al., 2018).

FILE S7 Annotated sequence of pCM1-029, the level 1 plasmid made up of the promoter AR (P_A/R_ + 5′UTR of RBCS2), *aadA* coding sequence and the terminator RBCS2 (3′UTR of RBCS2 + T_RBCS2_) conferring spectinomycin resistance in Chlamydomonas (Meslet-Cladière and Vallon, 2011; Crozet et al., 2018).
