# Supplementary figures and images for: Blasticidin S Deaminase: A New Efficient Selectable Marker for Chlamydomonas reinhardtii
Source: Front Plant Sci. 2020 Mar 5;11:242. doi: 10.3389/fpls.2020.00242 (PMC7066984; doi:10.3389/fpls.2020.00242)

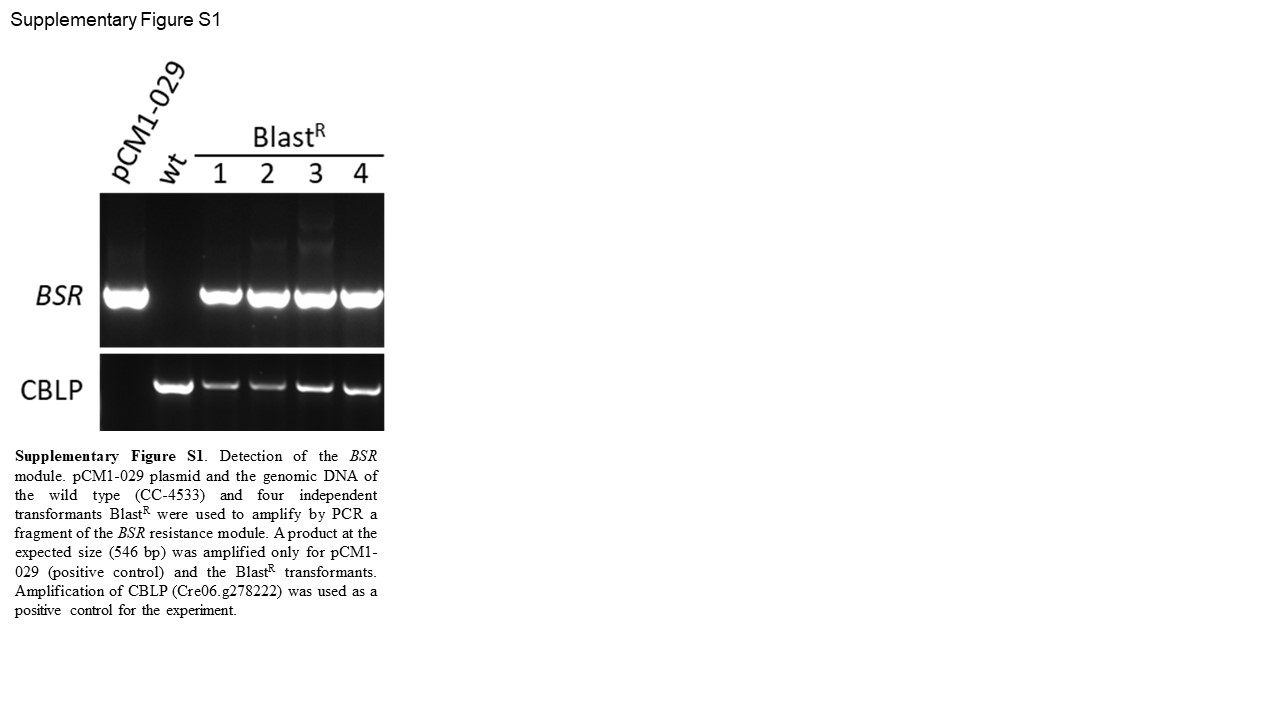

Supplement: FIGURE S1 — Detection of the BSR module. pCM1-029 plasmid and the genomic DNA of the wild type (CC-4533) and four independent transformants BlastR were used to amplify by PCR a fragment of the BSR resistance module. A product at the expected size (546 bp) was amplified only for pCM1-029 (positive control) and the BlastR transformants. Amplification of CBLP (Cre06.g278222) was used as a positive control for the experiment. [file Image_1.JPEG]

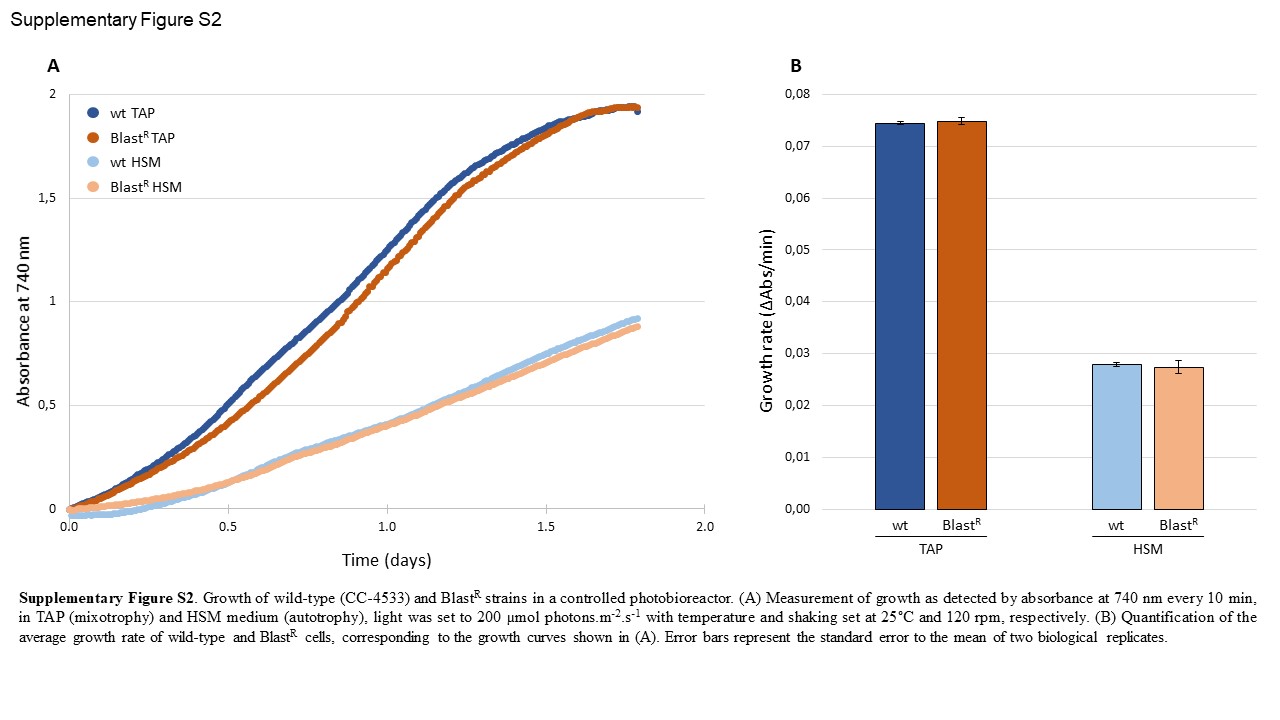

Supplement: FIGURE S2 — Growth of wild-type (CC-4533) and BlastR strains in a controlled photobioreactor. (A) Measurement of growth as detected by absorbance at 740 nm every 10 min, in TAP (mixotrophy) and HSM medium (autotrophy), light was set to 200 μmol photons⋅m–2⋅s–1 with temperature and shaking set at 25°C and 120 rpm, respectively. (B) Quantification of the average growth rate of wild-type and BlastR cells, corresponding to the growth curves shown in Graph (A). Error bars represent the standard error to the mean of two biological replicates. [file Image_2.JPEG]

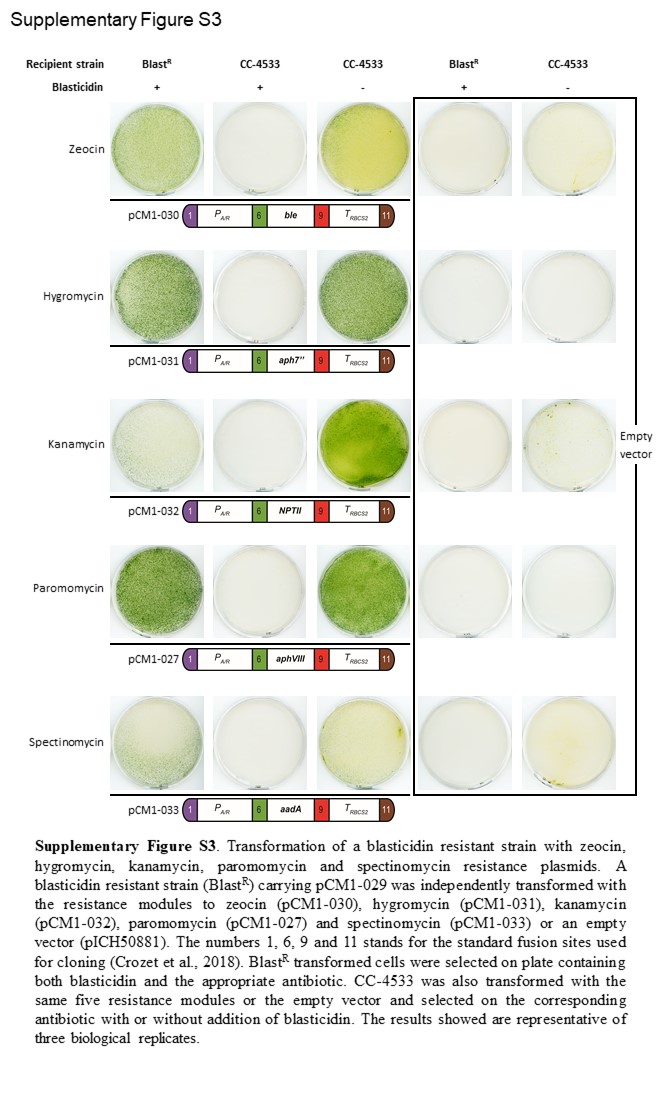

Supplement: FIGURE S3 — Transformation of a blasticidin resistant strain with zeocin, hygromycin, kanamycin, paromomycin and spectinomycin resistance plasmids. A blasticidin resistant strain (BlastR) carrying pCM1-029 was independently transformed with the resistance modules to zeocin (pCM1-030), hygromycin (pCM1-031), kanamycin (pCM1-032), paromomycin (pCM1-027), and spectinomycin (pCM1-033) or an empty vector (pICH50881). The numbers 1, 6, 9, and 11 stand for the standard fusion sites used for cloning (Crozet et al., 2018). BlastR transformed cells were selected on plate containing both blasticidin and the appropriate antibiotic. CC-4533 was also transformed with the same five resistance modules or the empty vector and selected on the corresponding antibiotic with or without addition of blasticidin. The results showed are representative of three biological replicates. [file Image_3.JPEG]
