# Supplementary material for: Blasticidin S Deaminase: A New Efficient Selectable Marker for Chlamydomonas reinhardtii
Source: Front Plant Sci. 2020 Mar 5;11:242. doi: 10.3389/fpls.2020.00242 (PMC7066984; doi:10.3389/fpls.2020.00242)
Supplement: FILE S1 — Annotated sequence of pCM0-120, the level 0 plasmid containing BSR coding sequence engineered for Chlamydomonas nuclear genome and designed for the position B3–B5 of the Chlamydomonas MoClo toolkit (Crozet et al., 2018). [file Data_Sheet_1.docx]

> [pCM0-120-BSR_(B3-B5).xdna - 2671 bp] Ligation of : pICH41308-pL0-CDS1.xdna [2243 nt] : (#BbsI[1438] / #BbsI[854]) to bc_bsr_synth.xdna [420 nt] : (#BbsI[3] / #BbsI[441])

aaaggatcttcttgagatcctttttttctgcgcgtaatctgctgcttgcaaacaaaaaaaccaccgctaccagcggtggt

ttgtttgccggatcaagagctaccaactctttttccgaaggtaactggcttcagcagagcgcagataccaaatactgtcc

ttctagtgtagccgtagttaggccaccacttcaagaactctgtagcaccgcctacatacctcgctctgctaatcctgtta

ccagtggctgctgccagtggcgataagtcgtgtcttaccgggttggactcaagacgatagttaccggataaggcgcagcg

gtcgggctgaacggggggttcgtgcacacagcccagcttggagcgaacgacctacaccgaactgagatacctacagcgtg

agctatgagaaagcgccacgcttcccgaagggagaaaggcggacaggtatccggtaagcggcagggtcggaacaggagag

cgcacgagggagcttccagggggaaacgcctggtatctttatagtcctgtcgggtttcgccacctctgacttgagcgtcg

atttttgtgatgctcgtcaggggggcggagcctatggaaaaacgccagcaacgcggcctttttacggttcctggcctttt

gctggccttttgctcacatgttctttcctgcgttatcccctgattctgtggataaccgtattaccgcctttgagtgagct

gataccgctcgccgcagccgaacgaccgagcgcagcgagtcagtgagcgaggaagcggaagagcgcccaatacgcaaacc

gcctctccccgcgcgttggccgattcattaatcactctgtggtctcaaatgAAAACCTTCAACATCAGCCAGCAGGACCT

GGAGCTGGTGGAGGTGGCCACCGAGAAGATCACCATGCTGTACGAGGACAACAAGCACCACGTGGGCGCCGCCATCCGCA

CCAAGACCGGCGAGATCATCAGCGCCGTGCACATCGAGGCCTACATCGGCCGCGTGACCGTGTGCGCCGAGGCCATCGCC

ATCGGCAGCGCCGTGAGCAACGGCCAGAAGGACTTCGACACCATCGTGGCCGTGCGCCACCCCTACAGCGACGAGGTGGA

CCGCAGCATCCGCGTGGTGAGCCCCTGCGGCATGTGCCGCGAGCTGATCAGCGACTACGCCCCCGACTGCTTCGTGCTGA

TCGAGATGAACGGCAAGCTGGTCAAGACCACCATCGAGGAGCTGATCCCCCTGAAGTACACCCGCAACTAAgctttgaga

ccacgaagtggctcttcagtggacgaaagggcctcgtgatacgcctatttttataggttaatgtcatgataataatggtt

tcttagacgtcaggtggcacttttcggggaaatgtgcgcggaacccctatttgtttatttttctaaatacattcaaatat

gtatccgctcatgagacaataaccctgataaatgcttcaataatattgaaaaaggaagagtatgcgctcacgcaactggt

ccagaaccttgaccgaacgcagcggtggtaacggcgcagtggcggttttcatggcttgttatgactgtttttttggggta

cagtctatgcctcgggcatccaagcagcaagcgcgttacgccgtgggtcgatgtttgatgttatggagcagcaacgatgt

tacgcagcagggcagtcgccctaaaacaaagttaaacatcatgagggaagcggtgatcgccgaagtatcgactcaactat

cagaggtagttggcgtcatcgagcgccatctcgaaccgacgttgctggccgtacatttgtacggctccgcagtggatggc

ggcctgaagccacacagcgatattgatttgctggttacggtgaccgtaaggcttgatgaaacaacgcggcgagctttgat

caacgaccttttggaaacttcggcttcccctggagagagcgagattctccgcgctgtagaagtcaccattgttgtgcacg

acgacatcattccgtggcgttatccagctaagcgcgaactgcaatttggagaatggcagcgcaatgacattcttgcaggt

atcttcgagccagccacgatcgacattgatctggctatcttgctgacaaaagcaagagaacatagcgttgccttggtagg

tccagcggcggaggaactctttgatccggttcctgaacaggatctatttgaggcgctaaatgaaaccttaacgctatgga

actcgccgcccgactgggctggcgatgagcgaaatgtagtgcttacgttgtcccgcatttggtacagcgcagtaaccggc

aaaatcgcgccgaaggatgtcgctgccgactgggcaatggagcgcctgccggcccagtatcagcccgtcatacttgaagc

tagacaggcttatcttggacaagaagaagatcgcttggcctcgcgcgcagatcagttggaagaatttgtccattacgtaa

aaggcgagatcaccaaggtagtcggcaaataactgtcagaccaagtttactcatatatactttagattgatttaaaactt

catttttaatttaaaaggatctaggtgaagatcctttttgataatctcatgaccaaaatcccttaacgtgagttttcgtt

ccactgagcgtcagaccccgtagaaaagatc

Features :

Sm/Sp\no\DraIII : [1502 : 2512 - CW]

G\to\A : [2479 : 2479 - CW]

BSR-BlastR : [849 : 1271 - CW]

ColE1 origin : [2661 : 618 - CW]

Amp prom : [1432 : 1460 - CW]

BsaI : [841 : 846 - CW]

BsaI : [1282 : 1277 - CCW]
