# Supplementary material for: Blasticidin S Deaminase: A New Efficient Selectable Marker for Chlamydomonas reinhardtii
Source: Front Plant Sci. 2020 Mar 5;11:242. doi: 10.3389/fpls.2020.00242 (PMC7066984; doi:10.3389/fpls.2020.00242)
Supplement: FILE S2 — Annotated sequence of pCM1-029, the level 1 plasmid made up of the promoter AR (PA/R + 5′UTR of RBCS2), BSR coding sequence and the terminator of RBCS2 (3′UTR of RBCS2 + TRBCS2) conferring blasticidin resistance in Chlamydomonas (Crozet et al., 2018). [file Data_Sheet_2.docx]

> [pCM1-029-pAR-BSR-tRbcS2.xdna - 5707 bp] Ligation of : pL1-2F (pICH47742).xdna [4368 nt] : (#BsaI[619] / #BsaI[35]) to Ligation #3 [1331 nt] : (Uncut 5'[0] / Uncut 3'[1331])

tctgtgaagacaagcaagaattcaagcttggagGAAGGGCCGCGACGGTTCGAGAACCGACTTGAGGGCGCCAAACGAGC

CCGAGCCGCCGTTGCGCCAGGCGAAACCAGAACCGTAGATTAATGCACTTGAGCTATTCATTGGAGCGATCTGCCGGGGA

CAGCGGGTCTGGCGTGCGCGCGATTGGAGATCGCAAATTACATATGTCTGCGTGACGGCGGGGAGCTCGCTGAGGCTTGA

CATGATTGGTGCGTATGTTTGTATGAAGCTACAGGACTGATTTGGCGGGCTATGAGGGCGGGGGAAGCTCTGGAAGGGCC

GCGATGGGGCGCGCGGCGTCCAGAAGGCGCCATACGGCCCGCTGGCGGCACCCATCCGGTATAAAAGCCCGCGACCCCGA

ACGGTGACCTCCACTTTCAGCGACAAACGAGCACTTATACATACGCGACTATTCTGCCGCTATACATAACCACTCAGCTA

GCGATCCCGGGCGCGCCAGAAGGAGCGCAGCCAAACCAGGATGATGTTTGATGGGGTATTTGAGCACTTGCAACCCTTAT

CCGGAAGCCCCCTGGCCCACAAAGGCTAGGCGCCAATGCAAGCAGTTCGCATGCAGCCCCTGGAGCGGTGCCCTCCTGAT

AAACCGGCCAGGGGGCCTATGTTCTTTACTTTTTTACAAGAGAAGTCACTCAACATCTTAAaatgAAAACCTTCAACATC

AGCCAGCAGGACCTGGAGCTGGTGGAGGTGGCCACCGAGAAGATCACCATGCTGTACGAGGACAACAAGCACCACGTGGG

CGCCGCCATCCGCACCAAGACCGGCGAGATCATCAGCGCCGTGCACATCGAGGCCTACATCGGCCGCGTGACCGTGTGCG

CCGAGGCCATCGCCATCGGCAGCGCCGTGAGCAACGGCCAGAAGGACTTCGACACCATCGTGGCCGTGCGCCACCCCTAC

AGCGACGAGGTGGACCGCAGCATCCGCGTGGTGAGCCCCTGCGGCATGTGCCGCGAGCTGATCAGCGACTACGCCCCCGA

CTGCTTCGTGCTGATCGAGATGAACGGCAAGCTGGTCAAGACCACCATCGAGGAGCTGATCCCCCTGAAGTACACCCGCA

ACTAAgcttccgctccgtgtaaatggAGGCGCTCGTTGATCTGAGCCTTGCCCCCTGACGAACGGCGGTGGATGGAAGAT

ACTGCTCTCAAGTGCTGAAGCGGTAGCTTAGCTCCCCGTTTCGTGCTGATCAGTCTTTTTCAACACGTAAAAAGCGGAGG

AGTTTTGCAATTTTGTTGGTTGTAACGATCCTCCGTTGATTTTGGCCTCTTTCTCCATGGGCGGGCTgggcgtatttgaa

gcggcgctactattgtcttctgcacgaagtggtttaaactatcagtgtttgacaggatatattggcgggtaaacctaaga

gaaaagagcgtttattagaataatcggatatttaaaagggcgtgaaaaggtttatccgttcgtccatttgtatgtgcatg

ccaaccacagggttccccagatcaggcgctggctgctgaacccccagccggaactgaccccacaaggccctagcgtttgc

aatgcaccaggtcatcattgacccaggcgtgttccaccaggccgctgcctcgcaactcttcgcaggcttcgccgacctgc

tcgcgccacttcttcacgcgggtggaatccgatccgcacatgaggcggaaggtttccagcttgagcgggtacggctcccg

gtgcgagctgaaatagtcgaacatccgtcgggccgtcggcgacagcttgcggtacttctcccatatgaatttcgtgtagt

ggtcgccagcaaacagcacgacgatttcctcgtcgatcaggacctggcaacgggacgttttcttgccacggtccaggacg

cggaagcggtgcagcagcgacaccgattccaggtgcccaacgcggtcggacgtgaagcccatcgccgtcgcctgtaggcg

cgacaggcattcctcggccttcgtgtaataccggccattgatcgaccagcccaggtcctggcaaagctcgtagaacgtga

aggtgatcggctcgccgataggggtgcgcttcgcgtactccaacacctgctgccacaccagttcgtcatcgtcggcccgc

agctcgacgccggtgtaggtgatcttcacgtccttgttgacgtggaaaatgaccttgttttgcagcgcctcgcgcgggat

tttcttgttgcgcgtggtgaacagggcagagcgggccgtgtcgtttggcatcgctcgcatcgtgtccggccacggcgcaa

tatcgaacaaggaaagctgcatttccttgatctgctgcttcgtgtgtttcagcaacgcggcctgcttggcctcgctgacc

tgttttgccaggtcctcgccggcggtttttcgcttcttggtcgtcatagttcctcgcgtgtcgatggtcatcgacttcgc

caaacctgccgcctcctgttcaagacgacgcgaacgctccacggcggccgatggcgcgggcagggcagggggagccagtt

gcacgctgtcgcgctcgatcttggccgtagcttgctggaccatcgagccgacggactggaaggtttcgcggggcgcacgc

atgacggtgcggcttgcgatggtttcggcatcctcggcggaaaaccccgcgtcgatcagttcttgcctgtatgccttccg

gtcaaacgtccgattcattcaccctccttgcgggattgccccgactcacgccggggcaatgtgcccttattcctgatttg

acccgcctggtgccttggtgtccagataatccaccttatcggcaatgaagtcggtcccgtagaccgtctggccgtccttc

tcgtacttggtattccgaatcttgccctgcacgaataccagcgaccccttgcccaaatacttgccgtgggcctcggcctg

agagccaaaacacttgatgcggaagaagtcggtgcgctcctgcttgtcgccggcatcgttgcgccacatctaggatctgc

caggaaccgtaaaaaggccgcgttgctggcgtttttccataggctccgcccccctgacgagcatcacaaaaatcgacgct

caagtcagaggtggcgaaacccgacaggactataaagataccaggcgtttccccctggaagctccctcgtgcgctctcct

gttccgaccctgccgcttaccggatacctgtccgcctttctcccttcgggaagcgtggcgctttctcatagctcacgctg

taggtatctcagttcggtgtaggtcgttcgctccaagctgggctgtgtgcacgaaccccccgttcagcccgaccgctgcg

ccttatccggtaactatcgtcttgagtccaacccggtaagacacgacttatcgccactggcagcagccactggtaacagg

attagcagagcgaggtatgtaggcggtgctacagagttcttgaagtggtggcctaactacggctacactagaaggacagt

atttggtatctgcgctctgctgaagccagttaccttcggaaaaagagttggtagctcttgatccggcaaacaaaccaccg

ctggtagcggtggtttttttgtttgcaagcagcagattacgcgcagaaaaaaaggatctcaagaagatcctttgatcttt

tctacggggtctgacgctcagtggaacgaaaactcacgttaagggattttggtcatgagattatcaaaaaggatcttcac

ctagatccttttaaattaaaaatgaagttttaaatcaatctaaagtatatatgagtaaacttggtctgacagttaccaat

gcttaatcagtgaggcacctatctcagcgatctgtctatttcgttcatccatagttgcctgactccccgtcgtgtagata

actacgatacgggagggcttaccatctggccccagtgctgcaatgataccgcgagaaccacgctcaccggctccagattt

atcagcaataaaccagccagccggaagggccgagcgcagaagtggtcctgcaactttatccgcctccatccagtctatta

attgttgccgggaagctagagtaagtagttcgccagttaatagtttgcgcaacgttgttgccattgctacaggcatcgtg

gtgtcacgctcgtcgtttggtatggcttcattcagctccggttcccaacgatcaaggcgagttacatgatcccccatgtt

gtgcaaaaaagcggttagctccttcggtcctccgatcgttgtcagaagtaagttggccgcagtgttatcactcatggtta

tggcagcactgcataattctcttactgtcatgccatccgtaagatgcttttctgtgactggtgagtactcaaccaagtca

ttctgagaatagtgtatgcggcgaccgagttgctcttgcccggcgtcaatacgggataataccgcgccacatagcagaac

tttaaaagtgctcatcattggaaaacgttcttcggggcgaaaactctcaaggatcttaccgctgttgagatccagttcga

tgtaacccactcgtgcacccaactgatcttcagcatcttttactttcaccagcgtttctgggtgagcaaaaacaggaagg

caaaatgccgcaaaaaagggaataagggcgacacggaaatgttgaatactcatactcttcctttttcaatattattgaag

catttatcagggttattgtctcatgagcggatacatatttgaatgtatttagaaaaataaacaaataggggttccgcgca

cgaattggccagcgctgccatttttggggtgaggccgttcgcggccgaggggcgcagcccctggggggatgggaggcccg

cgttagcgggccgggagggttcgagaagggggggcaccccccttcggcgtgcgcggtcacgcgcacagggcgcagccctg

gttaaaaacaaggtttataaatattggtttaaaagcaggttaaaagacaggttagcggtggccgaaaaacgggcggaaac

ccttgcaaatgctggattttctgcctgtggacagcccctcaaatgtcaataggtgcgcccctcatctgtcagcactctgc

ccctcaagtgtcaaggatcgcgcccctcatctgtcagtagtcgcgcccctcaagtgtcaataccgcagggcacttatccc

caggcttgtccacatcatctgtgggaaactcgcgtaaaatcaggcgttttcgccgatttgcgaggctggccagctccacg

tcgccggccgaaatcgagcctgcccctcatctgtcaacgccgcgccgggtgagtcggcccctcaagtgtcaacgtccgcc

cctcatctgtcagtgagggccaagttttccgcgaggtatccacaacgccggcggccgcggtgtctcgcacacggcttcga

cggcgtttctggcgcgtttgcagggccatagacggccgccagcccagcggcgagggcaaccagcccggtgagcgtcgcaa

aggagatcctgatctgactgatgggctgcctgtatcgagtggtgattttgtgccgagctgccggtcggggagctgttggc

tggctggtggcaggatatattgtggtgtaaacaaattgacgcttagacaacttaataacacattgcggacgtttttaatg

tactggggtggatgcagtgggccccac

Features :

RK2\trfa\(no\Esp3I) : [3028 : 1547 - CCW]

RB\short : [1523 : 1396 - CCW]

shows similarity to T-DNA left border: GenBank Accession Number J01825_TDNA-LB : [5538 : 5685 - CW]

shows similarity to GenBank Accession Number M20134_oriV : [5508 : 4891 - CCW]

pUC\ori : [3039 : 3828 - CW]

RNaseH cleavage point_ORI : [3074 : 3074 - CW]

AP\r : [4693 : 3836 - CCW]

tRbcS2 : [1130 : 1363 - CW]

pHsp70A : [34 : 482 - CW]

5UTR CrRbcS2 : [680 : 702 - CW]

pRbcS2 : [487 : 679 - CW]

ColE1 origin : [3684 : 3056 - CCW]

Amp prom : [4763 : 4735 - CCW]

BbsI : [6 : 11 - CW]

BbsI : [1380 : 1375 - CCW]

BSR-BlastR : [703 : 1125 - CW]
