# Supplementary material for: Blasticidin S Deaminase: A New Efficient Selectable Marker for Chlamydomonas reinhardtii
Source: Front Plant Sci. 2020 Mar 5;11:242. doi: 10.3389/fpls.2020.00242 (PMC7066984; doi:10.3389/fpls.2020.00242)
Supplement: FILE S3 — Annotated sequence of pCM1-027, the level 1 plasmid made up of the promoter AR (PA/R + 5′UTR of RBCS2), aphVIII coding sequence and the terminator of RBCS2 (3′UTR of RBCS2 + TRBCS2) conferring paromomycin resistance in Chlamydomonas (Sizova et al., 2001; Crozet et al., 2018). [file Data_Sheet_3.docx]

> [pCM1-027-pAR-AphVIII-tRbcS2.xdna - 5914 bp] Ligation of : pL1-1F (pICH47732).xdna [4368 nt] : (#BsaI[4929] / #BsaI[4345]) to Ligation #8 [1538 nt] : (Uncut 3'[1538] / Uncut 5'[0])

gccaggaaccgtaaaaaggccgcgttgctggcgtttttccataggctccgcccccctgacgagcatcacaaaaatcgacg

ctcaagtcagaggtggcgaaacccgacaggactataaagataccaggcgtttccccctggaagctccctcgtgcgctctc

ctgttccgaccctgccgcttaccggatacctgtccgcctttctcccttcgggaagcgtggcgctttctcatagctcacgc

tgtaggtatctcagttcggtgtaggtcgttcgctccaagctgggctgtgtgcacgaaccccccgttcagcccgaccgctg

cgccttatccggtaactatcgtcttgagtccaacccggtaagacacgacttatcgccactggcagcagccactggtaaca

ggattagcagagcgaggtatgtaggcggtgctacagagttcttgaagtggtggcctaactacggctacactagaaggaca

gtatttggtatctgcgctctgctgaagccagttaccttcggaaaaagagttggtagctcttgatccggcaaacaaaccac

cgctggtagcggtggtttttttgtttgcaagcagcagattacgcgcagaaaaaaaggatctcaagaagatcctttgatct

tttctacggggtctgacgctcagtggaacgaaaactcacgttaagggattttggtcatgagattatcaaaaaggatcttc

acctagatccttttaaattaaaaatgaagttttaaatcaatctaaagtatatatgagtaaacttggtctgacagttacca

atgcttaatcagtgaggcacctatctcagcgatctgtctatttcgttcatccatagttgcctgactccccgtcgtgtaga

taactacgatacgggagggcttaccatctggccccagtgctgcaatgataccgcgagaaccacgctcaccggctccagat

ttatcagcaataaaccagccagccggaagggccgagcgcagaagtggtcctgcaactttatccgcctccatccagtctat

taattgttgccgggaagctagagtaagtagttcgccagttaatagtttgcgcaacgttgttgccattgctacaggcatcg

tggtgtcacgctcgtcgtttggtatggcttcattcagctccggttcccaacgatcaaggcgagttacatgatcccccatg

ttgtgcaaaaaagcggttagctccttcggtcctccgatcgttgtcagaagtaagttggccgcagtgttatcactcatggt

tatggcagcactgcataattctcttactgtcatgccatccgtaagatgcttttctgtgactggtgagtactcaaccaagt

cattctgagaatagtgtatgcggcgaccgagttgctcttgcccggcgtcaatacgggataataccgcgccacatagcaga

actttaaaagtgctcatcattggaaaacgttcttcggggcgaaaactctcaaggatcttaccgctgttgagatccagttc

gatgtaacccactcgtgcacccaactgatcttcagcatcttttactttcaccagcgtttctgggtgagcaaaaacaggaa

ggcaaaatgccgcaaaaaagggaataagggcgacacggaaatgttgaatactcatactcttcctttttcaatattattga

agcatttatcagggttattgtctcatgagcggatacatatttgaatgtatttagaaaaataaacaaataggggttccgcg

cacgaattggccagcgctgccatttttggggtgaggccgttcgcggccgaggggcgcagcccctggggggatgggaggcc

cgcgttagcgggccgggagggttcgagaagggggggcaccccccttcggcgtgcgcggtcacgcgcacagggcgcagccc

tggttaaaaacaaggtttataaatattggtttaaaagcaggttaaaagacaggttagcggtggccgaaaaacgggcggaa

acccttgcaaatgctggattttctgcctgtggacagcccctcaaatgtcaataggtgcgcccctcatctgtcagcactct

gcccctcaagtgtcaaggatcgcgcccctcatctgtcagtagtcgcgcccctcaagtgtcaataccgcagggcacttatc

cccaggcttgtccacatcatctgtgggaaactcgcgtaaaatcaggcgttttcgccgatttgcgaggctggccagctcca

cgtcgccggccgaaatcgagcctgcccctcatctgtcaacgccgcgccgggtgagtcggcccctcaagtgtcaacgtccg

cccctcatctgtcagtgagggccaagttttccgcgaggtatccacaacgccggcggccgcggtgtctcgcacacggcttc

gacggcgtttctggcgcgtttgcagggccatagacggccgccagcccagcggcgagggcaaccagcccggtgagcgtcgc

aaaggagatcctgatctgactgatgggctgcctgtatcgagtggtgattttgtgccgagctgccggtcggggagctgttg

gctggctggtggcaggatatattgtggtgtaaacaaattgacgcttagacaacttaataacacattgcggacgtttttaa

tgtactggggtggatgcagtgggccccactctgtgaagacaatgccgaattcggatccggaggctgaggcttgacatgat

tggtgcgtatgtttgtatgaagctacaggactgatttggcgggctatgagggcgggggaagctctggaagggccgcgatg

gggcgcgcggcgtccagaaggcgccatacggcccgctggcggcacccatccggtataaaagcccgcgaccccgaacggtg

acctccactttcagcgacaaacgagcacttatacatacgcgactattctgccgctatacataaccactcagctagcttaa

gatcccatcaccggtgcatgccgggcgcgccagaaggagcgcagccaaaccaggatgatgtttgatggggtatttgagca

cttgcaacccttatccggaagccccctggcccacaaaggctaggcgccaatgcaagcagttcgcatgcagcccctggagc

ggtgccctcctgataaaccggccagggggcctatgttctttacttttttacaagagaagtcactcaacatcttaaaaatg

GACGATGCGTTGCGTGCACTGCGGGGTCGGTATCCCGGTTGTGAGTGGGTTGTTGTGGAGGATGGGGCCTCGGGGGCTGG

TGTTTATCGGCTTCGGGGTGGTGGGCGGGAGTTGTTTGTCAAGGTGGCAGCTCTGGGGGCCGGGGTGGGCTTGTTGGGTG

AGGCTGAGCGGCTGGTGTGGTTGGCGGAGGTGGGGATTCCCGTACCTCGTGTTGTGGAGGGTGGTGGGGACGAGAGGGTC

GCCTGGTTGGTCACCGAAGCGGTTCCGGGGCGTCCGGCCAGTGCGCGGTGGCCGCGGGAGCAGCGGCTGGACGTGGCGGT

GGCGCTCGCGGGGCTCGCTCGTTCGCTGCACGCGCTGGACTGGGAGCGGTGTCCGTTCGATCGCAGTCTCGCGGTGACGG

TGCCGCAGGCGGCCCGTGCTGTCGCTGAAGGGAGCGTCGACTTGGAGGATCTGGACGAGGAGCGGAAGGGGTGGTCGGGG

GAGCGGCTTCTCGCCGAGCTGGAGCGGACTCGGCCTGCGGACGAGGATCTGGCGGTTTGCCACGGTCACCTGTGCCCGGA

CAACGTGCTGCTCGACCCTCGTACCTGCGAGGTGACCGGGCTGATCGACGTGGGGCGGGTCGGCCGTGCGGACCGGCACT

CCGATCTCGCGCTGGTGCTGCGCGAGCTGGCCCACGAGGAGGACCCGTGGTTCGGGCCGGAGTGTTCCGCGGCGTTCCTG

CGGGAGTACGGGCGCGGGTGGGATGGGGCGGTATCGGAGGAAAAGCTGGCGTTTTACCGGCTGTTGGACGAGTTCTTCTG

AgcttccgctccgtgtaaatggAGGCGCTCGTTGATCTGAGCCTTGCCCCCTGACGAACGGCGGTGGATGGAAGATACTG

CTCTCAAGTGCTGAAGCGGTAGCTTAGCTCCCCGTTTCGTGCTGATCAGTCTTTTTCAACACGTAAAAAGCGGAGGAGTT

TTGCAATTTTGTTGGTTGTAACGATCCTCCGTTGATTTTGGCCTCTTTCTCCATGGGCGGGCTgggcgtatttgaagcgg

cgctgcaattgtcttctgcacgaagtggtttaaactatcagtgtttgacaggatatattggcgggtaaacctaagagaaa

agagcgtttattagaataatcggatatttaaaagggcgtgaaaaggtttatccgttcgtccatttgtatgtgcatgccaa

ccacagggttccccagatcaggcgctggctgctgaacccccagccggaactgaccccacaaggccctagcgtttgcaatg

caccaggtcatcattgacccaggcgtgttccaccaggccgctgcctcgcaactcttcgcaggcttcgccgacctgctcgc

gccacttcttcacgcgggtggaatccgatccgcacatgaggcggaaggtttccagcttgagcgggtacggctcccggtgc

gagctgaaatagtcgaacatccgtcgggccgtcggcgacagcttgcggtacttctcccatatgaatttcgtgtagtggtc

gccagcaaacagcacgacgatttcctcgtcgatcaggacctggcaacgggacgttttcttgccacggtccaggacgcgga

agcggtgcagcagcgacaccgattccaggtgcccaacgcggtcggacgtgaagcccatcgccgtcgcctgtaggcgcgac

aggcattcctcggccttcgtgtaataccggccattgatcgaccagcccaggtcctggcaaagctcgtagaacgtgaaggt

gatcggctcgccgataggggtgcgcttcgcgtactccaacacctgctgccacaccagttcgtcatcgtcggcccgcagct

cgacgccggtgtaggtgatcttcacgtccttgttgacgtggaaaatgaccttgttttgcagcgcctcgcgcgggattttc

ttgttgcgcgtggtgaacagggcagagcgggccgtgtcgtttggcatcgctcgcatcgtgtccggccacggcgcaatatc

gaacaaggaaagctgcatttccttgatctgctgcttcgtgtgtttcagcaacgcggcctgcttggcctcgctgacctgtt

ttgccaggtcctcgccggcggtttttcgcttcttggtcgtcatagttcctcgcgtgtcgatggtcatcgacttcgccaaa

cctgccgcctcctgttcaagacgacgcgaacgctccacggcggccgatggcgcgggcagggcagggggagccagttgcac

gctgtcgcgctcgatcttggccgtagcttgctggaccatcgagccgacggactggaaggtttcgcggggcgcacgcatga

cggtgcggcttgcgatggtttcggcatcctcggcggaaaaccccgcgtcgatcagttcttgcctgtatgccttccggtca

aacgtccgattcattcaccctccttgcgggattgccccgactcacgccggggcaatgtgcccttattcctgatttgaccc

gcctggtgccttggtgtccagataatccaccttatcggcaatgaagtcggtcccgtagaccgtctggccgtccttctcgt

acttggtattccgaatcttgccctgcacgaataccagcgaccccttgcccaaatacttgccgtgggcctcggcctgagag

ccaaaacacttgatgcggaagaagtcggtgcgctcctgcttgtcgccggcatcgttgcgccacatctaggatct

Features :

RK2\trfa\(no\Esp3I) : [5904 : 4423 - CCW]

RB\short : [4399 : 4272 - CCW]

shows similarity to T-DNA left border: GenBank Accession Number J01825_TDNA-LB : [2500 : 2647 - CW]

shows similarity to GenBank Accession Number M20134_oriV : [2470 : 1853 - CCW]

pUC\ori : [1 : 790 - CW]

AP\r : [1655 : 798 - CCW]

AphVIII-ParoR : [3201 : 4001 - CW]

tRbcS2 : [4006 : 4239 - CW]

pHsp70A : [2703 : 2969 - CW]

5UTR CrRbcS2 : [3174 : 3196 - CW]

pRbcS2 : [2981 : 3173 - CW]

ColE1 origin : [646 : 18 - CCW]

Amp prom : [1725 : 1697 - CCW]

BbsI : [2675 : 2680 - CW]

BbsI : [4256 : 4251 - CCW]
