# Supplementary material for: Blasticidin S Deaminase: A New Efficient Selectable Marker for Chlamydomonas reinhardtii
Source: Front Plant Sci. 2020 Mar 5;11:242. doi: 10.3389/fpls.2020.00242 (PMC7066984; doi:10.3389/fpls.2020.00242)
Supplement: FILE S4 — Annotated sequence of pCM1-030, the level 1 plasmid made up of the promoter AR (PA/R + 5′UTR of RBCS2), Ble coding sequence and the terminator RBCS2 (3′UTR of RBCS2 + TRBCS2) conferring zeocin resistance in Chlamydomonas (Stevens et al., 1996; Crozet et al., 2018). [file Data_Sheet_4.docx]

> [pCM1-030-pAR-Ble-tRbcS2.xdna - 5636 bp] Ligation of : pL1-1F (pICH47732).xdna [4368 nt] : (#BsaI[619] / #BsaI[35]) to Ligation #1 [1260 nt] : (Uncut 5'[0] / Uncut 3'[1260])

tctgtgaagacaatgccgaattcggatccggaggctgaggcttgacatgattggtgcgtatgtttgtatgaagctacagg

actgatttggcgggctatgagggcgggggaagctctggaagggccgcgatggggcgcgcggcgtccagaaggcgccatac

ggcccgctggcggcacccatccggtataaaagcccgcgaccccgaacggtgacctccactttcagcgacaaacgagcact

tatacatacgcgactattctgccgctatacataaccactcagctagcttaagatcccatcaagcttgcatgccgggcgcg

ccagaaggagcgcagccaaaccaggatgatgtttgatggggtatttgagcacttgcaacccttatccggaagccccctgg

cccacaaaggctaggcgccaatgcaagcagttcgcatgcagcccctggagcggtgccctcctgataaaccggccaggggg

cctatgttctttacttttttacaagagaagtcactcaacatcttaaaaatgGCCAAGCTGACCAGCGCCGTTCCGGTGCT

CACCGCGCGCGACGTCGCCGGAGCGGTCGAGTTCTGGACCGACCGGCTCGGGTTCTCCCGGGACTTCGTGGAGGACGACT

TCGCCGGTGTGGTCCGGGACGACGTGACCCTGTTCATCAGCGCGGTCCAGGACCAGGTGAGTCGACGAGCAAGCCCGGCG

GATCAGGCAGCGTGCTTGCAGATTTGACTTGCAACGCCCGCATTGTGTCGACGAAGGCTTTTGGCTCCTCTGTCGCTGTC

TCAAGCAGCATCTAACCCTGCGTCGCCGTTTCCATTTGCAGGACCAGGTGGTGCCGGACAACACCCTGGCCTGGGTGTGG

GTGCGCGGCCTGGACGAGCTGTACGCCGAGTGGTCGGAGGTCGTGTCCACGAACTTCCGGGACGCCTCCGGGCCGGCCAT

GACCGAGATCGGCGAGCAGCCGTGGGGGCGGGAGTTCGCCCTGCGCGACCCGGCCGGCAACTGCGTGCACTTCGTGGCCG

AGGAGCAGGACTAAgcttccgctccgtgtaaatggAGGCGCTCGTTGATCTGAGCCTTGCCCCCTGACGAACGGCGGTGG

ATGGAAGATACTGCTCTCAAGTGCTGAAGCGGTAGCTTAGCTCCCCGTTTCGTGCTGATCAGTCTTTTTCAACACGTAAA

AAGCGGAGGAGTTTTGCAATTTTGTTGGTTGTAACGATCCTCCGTTGATTTTGGCCTCTTTCTCCATGGGCGGGCTgggc

gtatttgaagcggcgctgcaattgtcttctgcacgaagtggtttaaactatcagtgtttgacaggatatattggcgggta

aacctaagagaaaagagcgtttattagaataatcggatatttaaaagggcgtgaaaaggtttatccgttcgtccatttgt

atgtgcatgccaaccacagggttccccagatcaggcgctggctgctgaacccccagccggaactgaccccacaaggccct

agcgtttgcaatgcaccaggtcatcattgacccaggcgtgttccaccaggccgctgcctcgcaactcttcgcaggcttcg

ccgacctgctcgcgccacttcttcacgcgggtggaatccgatccgcacatgaggcggaaggtttccagcttgagcgggta

cggctcccggtgcgagctgaaatagtcgaacatccgtcgggccgtcggcgacagcttgcggtacttctcccatatgaatt

tcgtgtagtggtcgccagcaaacagcacgacgatttcctcgtcgatcaggacctggcaacgggacgttttcttgccacgg

tccaggacgcggaagcggtgcagcagcgacaccgattccaggtgcccaacgcggtcggacgtgaagcccatcgccgtcgc

ctgtaggcgcgacaggcattcctcggccttcgtgtaataccggccattgatcgaccagcccaggtcctggcaaagctcgt

agaacgtgaaggtgatcggctcgccgataggggtgcgcttcgcgtactccaacacctgctgccacaccagttcgtcatcg

tcggcccgcagctcgacgccggtgtaggtgatcttcacgtccttgttgacgtggaaaatgaccttgttttgcagcgcctc

gcgcgggattttcttgttgcgcgtggtgaacagggcagagcgggccgtgtcgtttggcatcgctcgcatcgtgtccggcc

acggcgcaatatcgaacaaggaaagctgcatttccttgatctgctgcttcgtgtgtttcagcaacgcggcctgcttggcc

tcgctgacctgttttgccaggtcctcgccggcggtttttcgcttcttggtcgtcatagttcctcgcgtgtcgatggtcat

cgacttcgccaaacctgccgcctcctgttcaagacgacgcgaacgctccacggcggccgatggcgcgggcagggcagggg

gagccagttgcacgctgtcgcgctcgatcttggccgtagcttgctggaccatcgagccgacggactggaaggtttcgcgg

ggcgcacgcatgacggtgcggcttgcgatggtttcggcatcctcggcggaaaaccccgcgtcgatcagttcttgcctgta

tgccttccggtcaaacgtccgattcattcaccctccttgcgggattgccccgactcacgccggggcaatgtgcccttatt

cctgatttgacccgcctggtgccttggtgtccagataatccaccttatcggcaatgaagtcggtcccgtagaccgtctgg

ccgtccttctcgtacttggtattccgaatcttgccctgcacgaataccagcgaccccttgcccaaatacttgccgtgggc

ctcggcctgagagccaaaacacttgatgcggaagaagtcggtgcgctcctgcttgtcgccggcatcgttgcgccacatct

aggatctgccaggaaccgtaaaaaggccgcgttgctggcgtttttccataggctccgcccccctgacgagcatcacaaaa

atcgacgctcaagtcagaggtggcgaaacccgacaggactataaagataccaggcgtttccccctggaagctccctcgtg

cgctctcctgttccgaccctgccgcttaccggatacctgtccgcctttctcccttcgggaagcgtggcgctttctcatag

ctcacgctgtaggtatctcagttcggtgtaggtcgttcgctccaagctgggctgtgtgcacgaaccccccgttcagcccg

accgctgcgccttatccggtaactatcgtcttgagtccaacccggtaagacacgacttatcgccactggcagcagccact

ggtaacaggattagcagagcgaggtatgtaggcggtgctacagagttcttgaagtggtggcctaactacggctacactag

aaggacagtatttggtatctgcgctctgctgaagccagttaccttcggaaaaagagttggtagctcttgatccggcaaac

aaaccaccgctggtagcggtggtttttttgtttgcaagcagcagattacgcgcagaaaaaaaggatctcaagaagatcct

ttgatcttttctacggggtctgacgctcagtggaacgaaaactcacgttaagggattttggtcatgagattatcaaaaag

gatcttcacctagatccttttaaattaaaaatgaagttttaaatcaatctaaagtatatatgagtaaacttggtctgaca

gttaccaatgcttaatcagtgaggcacctatctcagcgatctgtctatttcgttcatccatagttgcctgactccccgtc

gtgtagataactacgatacgggagggcttaccatctggccccagtgctgcaatgataccgcgagaaccacgctcaccggc

tccagatttatcagcaataaaccagccagccggaagggccgagcgcagaagtggtcctgcaactttatccgcctccatcc

agtctattaattgttgccgggaagctagagtaagtagttcgccagttaatagtttgcgcaacgttgttgccattgctaca

ggcatcgtggtgtcacgctcgtcgtttggtatggcttcattcagctccggttcccaacgatcaaggcgagttacatgatc

ccccatgttgtgcaaaaaagcggttagctccttcggtcctccgatcgttgtcagaagtaagttggccgcagtgttatcac

tcatggttatggcagcactgcataattctcttactgtcatgccatccgtaagatgcttttctgtgactggtgagtactca

accaagtcattctgagaatagtgtatgcggcgaccgagttgctcttgcccggcgtcaatacgggataataccgcgccaca

tagcagaactttaaaagtgctcatcattggaaaacgttcttcggggcgaaaactctcaaggatcttaccgctgttgagat

ccagttcgatgtaacccactcgtgcacccaactgatcttcagcatcttttactttcaccagcgtttctgggtgagcaaaa

acaggaaggcaaaatgccgcaaaaaagggaataagggcgacacggaaatgttgaatactcatactcttcctttttcaata

ttattgaagcatttatcagggttattgtctcatgagcggatacatatttgaatgtatttagaaaaataaacaaatagggg

ttccgcgcacgaattggccagcgctgccatttttggggtgaggccgttcgcggccgaggggcgcagcccctggggggatg

ggaggcccgcgttagcgggccgggagggttcgagaagggggggcaccccccttcggcgtgcgcggtcacgcgcacagggc

gcagccctggttaaaaacaaggtttataaatattggtttaaaagcaggttaaaagacaggttagcggtggccgaaaaacg

ggcggaaacccttgcaaatgctggattttctgcctgtggacagcccctcaaatgtcaataggtgcgcccctcatctgtca

gcactctgcccctcaagtgtcaaggatcgcgcccctcatctgtcagtagtcgcgcccctcaagtgtcaataccgcagggc

acttatccccaggcttgtccacatcatctgtgggaaactcgcgtaaaatcaggcgttttcgccgatttgcgaggctggcc

agctccacgtcgccggccgaaatcgagcctgcccctcatctgtcaacgccgcgccgggtgagtcggcccctcaagtgtca

acgtccgcccctcatctgtcagtgagggccaagttttccgcgaggtatccacaacgccggcggccgcggtgtctcgcaca

cggcttcgacggcgtttctggcgcgtttgcagggccatagacggccgccagcccagcggcgagggcaaccagcccggtga

gcgtcgcaaaggagatcctgatctgactgatgggctgcctgtatcgagtggtgattttgtgccgagctgccggtcgggga

gctgttggctggctggtggcaggatatattgtggtgtaaacaaattgacgcttagacaacttaataacacattgcggacg

tttttaatgtactggggtggatgcagtgggccccac

Features :

RK2\trfa\(no\Esp3I) : [2957 : 1476 - CCW]

RB\short : [1452 : 1325 - CCW]

shows similarity to T-DNA left border: GenBank Accession Number J01825_TDNA-LB : [5467 : 5614 - CW]

shows similarity to GenBank Accession Number M20134_oriV : [5437 : 4820 - CCW]

pUC\ori : [2968 : 3757 - CW]

RNaseH cleavage point_ORI : [3003 : 3003 - CW]

AP\r : [4622 : 3765 - CCW]

Ble(i)-BleoR : [529 : 1054 - CW]

P-HSP70A : [34 : 300 - CW]

T-RBCS2 : [1059 : 1292 - CW]

CrRbcS2 intron 1 : [697 : 841 - CW]

5UTR CrRBCS2 : [505 : 527 - CW]

P-RBCS2 : [312 : 504 - CW]

BleomycinR-Nter : [529 : 696 - CW]

BleomycinR-Cter : [842 : 1051 - CW]

ColE1 origin : [3613 : 2985 - CCW]

Amp prom : [4692 : 4664 - CCW]

BbsI : [6 : 11 - CW]

BbsI : [1309 : 1304 - CCW]
