# Supplementary material for: Blasticidin S Deaminase: A New Efficient Selectable Marker for Chlamydomonas reinhardtii
Source: Front Plant Sci. 2020 Mar 5;11:242. doi: 10.3389/fpls.2020.00242 (PMC7066984; doi:10.3389/fpls.2020.00242)
Supplement: FILE S5 — Annotated sequence of pCM1-031, the level 1 plasmid made up of the promoter AR (PA/R + 5′UTR of RBCS2), aph7″ coding sequence and the terminator RBCS2 (3′UTR of RBCS2 + TRBCS2) conferring hygromycin resistance in Chlamydomonas (Berthold et al., 2002; Crozet et al., 2018). [file Data_Sheet_5.docx]

> [pCM1-031-pAR-aph7''-tRbcS2.xdna - 6109 bp] Ligation of : pL1-1F (pICH47732).xdna [4368 nt] : (#BsaI[619] / #BsaI[35]) to Ligation #2 [1733 nt] : (Uncut 5'[0] / Uncut 3'[1733])

gccaggaaccgtaaaaaggccgcgttgctggcgtttttccataggctccgcccccctgacgagcatcacaaaaatcgacg

ctcaagtcagaggtggcgaaacccgacaggactataaagataccaggcgtttccccctggaagctccctcgtgcgctctc

ctgttccgaccctgccgcttaccggatacctgtccgcctttctcccttcgggaagcgtggcgctttctcatagctcacgc

tgtaggtatctcagttcggtgtaggtcgttcgctccaagctgggctgtgtgcacgaaccccccgttcagcccgaccgctg

cgccttatccggtaactatcgtcttgagtccaacccggtaagacacgacttatcgccactggcagcagccactggtaaca

ggattagcagagcgaggtatgtaggcggtgctacagagttcttgaagtggtggcctaactacggctacactagaaggaca

gtatttggtatctgcgctctgctgaagccagttaccttcggaaaaagagttggtagctcttgatccggcaaacaaaccac

cgctggtagcggtggtttttttgtttgcaagcagcagattacgcgcagaaaaaaaggatctcaagaagatcctttgatct

tttctacggggtctgacgctcagtggaacgaaaactcacgttaagggattttggtcatgagattatcaaaaaggatcttc

acctagatccttttaaattaaaaatgaagttttaaatcaatctaaagtatatatgagtaaacttggtctgacagttacca

atgcttaatcagtgaggcacctatctcagcgatctgtctatttcgttcatccatagttgcctgactccccgtcgtgtaga

taactacgatacgggagggcttaccatctggccccagtgctgcaatgataccgcgagaaccacgctcaccggctccagat

ttatcagcaataaaccagccagccggaagggccgagcgcagaagtggtcctgcaactttatccgcctccatccagtctat

taattgttgccgggaagctagagtaagtagttcgccagttaatagtttgcgcaacgttgttgccattgctacaggcatcg

tggtgtcacgctcgtcgtttggtatggcttcattcagctccggttcccaacgatcaaggcgagttacatgatcccccatg

ttgtgcaaaaaagcggttagctccttcggtcctccgatcgttgtcagaagtaagttggccgcagtgttatcactcatggt

tatggcagcactgcataattctcttactgtcatgccatccgtaagatgcttttctgtgactggtgagtactcaaccaagt

cattctgagaatagtgtatgcggcgaccgagttgctcttgcccggcgtcaatacgggataataccgcgccacatagcaga

actttaaaagtgctcatcattggaaaacgttcttcggggcgaaaactctcaaggatcttaccgctgttgagatccagttc

gatgtaacccactcgtgcacccaactgatcttcagcatcttttactttcaccagcgtttctgggtgagcaaaaacaggaa

ggcaaaatgccgcaaaaaagggaataagggcgacacggaaatgttgaatactcatactcttcctttttcaatattattga

agcatttatcagggttattgtctcatgagcggatacatatttgaatgtatttagaaaaataaacaaataggggttccgcg

cacgaattggccagcgctgccatttttggggtgaggccgttcgcggccgaggggcgcagcccctggggggatgggaggcc

cgcgttagcgggccgggagggttcgagaagggggggcaccccccttcggcgtgcgcggtcacgcgcacagggcgcagccc

tggttaaaaacaaggtttataaatattggtttaaaagcaggttaaaagacaggttagcggtggccgaaaaacgggcggaa

acccttgcaaatgctggattttctgcctgtggacagcccctcaaatgtcaataggtgcgcccctcatctgtcagcactct

gcccctcaagtgtcaaggatcgcgcccctcatctgtcagtagtcgcgcccctcaagtgtcaataccgcagggcacttatc

cccaggcttgtccacatcatctgtgggaaactcgcgtaaaatcaggcgttttcgccgatttgcgaggctggccagctcca

cgtcgccggccgaaatcgagcctgcccctcatctgtcaacgccgcgccgggtgagtcggcccctcaagtgtcaacgtccg

cccctcatctgtcagtgagggccaagttttccgcgaggtatccacaacgccggcggccgcggtgtctcgcacacggcttc

gacggcgtttctggcgcgtttgcagggccatagacggccgccagcccagcggcgagggcaaccagcccggtgagcgtcgc

aaaggagatcctgatctgactgatgggctgcctgtatcgagtggtgattttgtgccgagctgccggtcggggagctgttg

gctggctggtggcaggatatattgtggtgtaaacaaattgacgcttagacaacttaataacacattgcggacgtttttaa

tgtactggggtggatgcagtgggccccactctgtgaagacaatgccgaattcggatccggaggctgaggcttgacatgat

tggtgcgtatgtttgtatgaagctacaggactgatttggcgggctatgagggcgggggaagctctggaagggccgcgatg

gggcgcgcggcgtccagaaggcgccatacggcccgctggcggcacccatccggtataaaagcccgcgaccccgaacggtg

acctccactttcagcgacaaacgagcacttatacatacgcgactattctgccgctatacataaccactcagctagcttaa

gatcccatcaagcttgcatgccgggcgcgccagaaggagcgcagccaaaccaggatgatgtttgatggggtatttgagca

cttgcaacccttatccggaagccccctggcccacaaaggctaggcgccaatgcaagcagttcgcatgcagcccctggagc

ggtgccctcctgataaaccggccagggggcctatgttctttacttttttacaagagaagtcactcaacatcttaaaaatg

acacaagaatccctgttacttctcgaccgtattgattcggatgattcctacgcgagcctgcggaacgaccaggagttctg

ggagccgctggcccgccgagccctggaggagctcgggctgccggtgccgccggtgctgcgggtgcccggcgagagcacca

accccgtactggtcggcgagcccggcccggtgatcaagctgttcggcgagcactggtgcggtccggagagcctcgcgtcg

gagtcggaggcctacgcggtcctggcggacgccccggtgccggtgccccgcctcctcggccgcggcgagctgcggcccgg

caccggagcctggccgtggccctacctggtgatgagccggatgaccggcaccacctggcggtccgcgatggacggcacga

ccgaccggaacgcgctgctcgccctggcccgcgaactcggccgggtgctcggccggctgcacagggtgccgctgaccggg

aacaccgtgctcaccccccattccgaggtgttcccggaactgctgcgggaacgccgcgcggcgaccgtcgaggaccaccg

cgggtggggctacctctcgccccggctgctggaccgcctggaggactggctgccggacgtggacacgctgctggccggcc

gcgaaccccggttcgtccacggcgacctgcacgggaccaacatcttcgtggacctggccgcgaccgaggtcaccgggatc

gtcgacttcaccgacgtgtatgcgggagactcccgctacagcctggtgcaactgcatctcaacgccttccggggcgaccg

cgagatcctggccgcgctgctcgacggggcgcagtggaagcggaccgaggacttcgcccgcgaactgctcgccttcacct

tcctgcacgacttcgaggtgttcgaggagacgccgctggatctctccggcttcaccgatccggaggaactggcgcagttc

ctctgggggccgccggacaccgcccccggcgcctgagcttccgctccgtgtaaatggAGGCGCTCGTTGATCTGAGCCTT

GCCCCCTGACGAACGGCGGTGGATGGAAGATACTGCTCTCAAGTGCTGAAGCGGTAGCTTAGCTCCCCGTTTCGTGCTGA

TCAGTCTTTTTCAACACGTAAAAAGCGGAGGAGTTTTGCAATTTTGTTGGTTGTAACGATCCTCCGTTGATTTTGGCCTC

TTTCTCCATGGGCGGGCTgggcgtatttgaagcggcgctgcaattgtcttctgcacgaagtggtttaaactatcagtgtt

tgacaggatatattggcgggtaaacctaagagaaaagagcgtttattagaataatcggatatttaaaagggcgtgaaaag

gtttatccgttcgtccatttgtatgtgcatgccaaccacagggttccccagatcaggcgctggctgctgaacccccagcc

ggaactgaccccacaaggccctagcgtttgcaatgcaccaggtcatcattgacccaggcgtgttccaccaggccgctgcc

tcgcaactcttcgcaggcttcgccgacctgctcgcgccacttcttcacgcgggtggaatccgatccgcacatgaggcgga

aggtttccagcttgagcgggtacggctcccggtgcgagctgaaatagtcgaacatccgtcgggccgtcggcgacagcttg

cggtacttctcccatatgaatttcgtgtagtggtcgccagcaaacagcacgacgatttcctcgtcgatcaggacctggca

acgggacgttttcttgccacggtccaggacgcggaagcggtgcagcagcgacaccgattccaggtgcccaacgcggtcgg

acgtgaagcccatcgccgtcgcctgtaggcgcgacaggcattcctcggccttcgtgtaataccggccattgatcgaccag

cccaggtcctggcaaagctcgtagaacgtgaaggtgatcggctcgccgataggggtgcgcttcgcgtactccaacacctg

ctgccacaccagttcgtcatcgtcggcccgcagctcgacgccggtgtaggtgatcttcacgtccttgttgacgtggaaaa

tgaccttgttttgcagcgcctcgcgcgggattttcttgttgcgcgtggtgaacagggcagagcgggccgtgtcgtttggc

atcgctcgcatcgtgtccggccacggcgcaatatcgaacaaggaaagctgcatttccttgatctgctgcttcgtgtgttt

cagcaacgcggcctgcttggcctcgctgacctgttttgccaggtcctcgccggcggtttttcgcttcttggtcgtcatag

ttcctcgcgtgtcgatggtcatcgacttcgccaaacctgccgcctcctgttcaagacgacgcgaacgctccacggcggcc

gatggcgcgggcagggcagggggagccagttgcacgctgtcgcgctcgatcttggccgtagcttgctggaccatcgagcc

gacggactggaaggtttcgcggggcgcacgcatgacggtgcggcttgcgatggtttcggcatcctcggcggaaaaccccg

cgtcgatcagttcttgcctgtatgccttccggtcaaacgtccgattcattcaccctccttgcgggattgccccgactcac

gccggggcaatgtgcccttattcctgatttgacccgcctggtgccttggtgtccagataatccaccttatcggcaatgaa

gtcggtcccgtagaccgtctggccgtccttctcgtacttggtattccgaatcttgccctgcacgaataccagcgacccct

tgcccaaatacttgccgtgggcctcggcctgagagccaaaacacttgatgcggaagaagtcggtgcgctcctgcttgtcg

ccggcatcgttgcgccacatctaggatct

Features :

RK2\trfa\(no\Esp3I) : [6099 : 4618 - CCW]

RB\short : [4594 : 4467 - CCW]

shows similarity to T-DNA left border: GenBank Accession Number J01825_TDNA-LB : [2500 : 2647 - CW]

shows similarity to GenBank Accession Number M20134_oriV : [2470 : 1853 - CCW]

pUC\ori : [1 : 790 - CW]

RNaseH cleavage point_ORI : [36 : 36 - CW]

AP\r : [1655 : 798 - CCW]

aph7''-HygroR : [3198 : 4196 - CW]

pHsp70A : [2703 : 2969 - CW]

tRbcS2 : [4201 : 4434 - CW]

5UTR CrRbcS2 : [3174 : 3196 - CW]

pRbcS2 : [2981 : 3173 - CW]

ColE1 origin : [646 : 18 - CCW]

Amp prom : [1725 : 1697 - CCW]

pBla with RBS : [1656 : 1725 - CW]

BbsI site : [2675 : 2680 - CW]

BbsI site : [4451 : 4446 - CCW]
