# Supplementary material for: Blasticidin S Deaminase: A New Efficient Selectable Marker for Chlamydomonas reinhardtii
Source: Front Plant Sci. 2020 Mar 5;11:242. doi: 10.3389/fpls.2020.00242 (PMC7066984; doi:10.3389/fpls.2020.00242)
Supplement: FILE S6 — Annotated sequence of pCM1-032, the level 1 plasmid made up of the promoter AR (PA/R + 5′UTR of RBCS2), NPTII coding sequence and the terminator RBCS2 (3′UTR of RBCS2 + TRBCS2) conferring kanamycin resistance in Chlamydomonas (Barahimipour et al., 2016; Crozet et al., 2018). [file Data_Sheet_6.docx]

> [pCM1-032-pAR-NPTII-tRbcS2.xdna - 5902 bp] Ligation of : pL1-1F (pICH47732).xdna [4368 nt] : (#BsaI[619] / #BsaI[35]) to Ligation #1 [1526 nt] : (Uncut 5'[0] / Uncut 3'[1526])

tctgtgaagacaatgccgaattcggatccggaggctgaggcttgacatgattggtgcgtatgtttgtatgaagctacagg

actgatttggcgggctatgagggcgggggaagctctggaagggccgcgatggggcgcgcggcgtccagaaggcgccatac

ggcccgctggcggcacccatccggtataaaagcccgcgaccccgaacggtgacctccactttcagcgacaaacgagcact

tatacatacgcgactattctgccgctatacataaccactcagctagcttaagatcccatcaagcttgcatgccgggcgcg

ccagaaggagcgcagccaaaccaggatgatgtttgatggggtatttgagcacttgcaacccttatccggaagccccctgg

cccacaaaggctaggcgccaatgcaagcagttcgcatgcagcccctggagcggtgccctcctgataaaccggccaggggg

cctatgttctttacttttttacaagagaagtcactcaacatcttaaaaatggagcaggacggcctgcacgccggcagccc

cgccgcctgggtggagcgcctgttcggctacgactgggcccagcagaccatcggctgcagcgacgccgccgtgttccgcc

tgagcgcccagggccgccccgtgctgttcgtcaagaccgacctgagcggcgccctgaacgagctgcaggacgaggccgcc

cgcctgagctggctggccaccaccggcgtgccctgcgccgccgtgctggacgtggtgaccgaggccggccgcgactggct

gctgctgggcgaggtgcccggccaggacctgctgagcagccacctggcccccgccgagaaggtgagcatcatggccgacg

ccatgcgccgcctgcacaccctggaccccgccacctgccccttcgaccaccaggccaagcaccgcatcgagcgcgcccgc

acccgcatggaggccggcctggtggaccaggacgacctggacgaggagcaccagggcctggcccccgccgagctgttcgc

ccgcctgaaggcccgcatgcccgacggcgaggacctggtggtgacccacggcgacgcctgcctgcccaacatcatggtgg

agaacggccgcttcagcggcttcatcgactgcggccgcctgggcgtggccgaccgctaccaggacatcgccctggccacc

cgcgacatcgccgaggagctgggcggcgagtgggccgaccgcttcctggtgctgtacggcatcgccgcccccgacagcca

gcgcatcgccttctaccgcctgctggacgagttcttctaagcttccgctccgtgtaaatggAGGCGCTCGTTGATCTGAG

CCTTGCCCCCTGACGAACGGCGGTGGATGGAAGATACTGCTCTCAAGTGCTGAAGCGGTAGCTTAGCTCCCCGTTTCGTG

CTGATCAGTCTTTTTCAACACGTAAAAAGCGGAGGAGTTTTGCAATTTTGTTGGTTGTAACGATCCTCCGTTGATTTTGG

CCTCTTTCTCCATGGGCGGGCTgggcgtatttgaagcggcgctgcaattgtcttctgcacgaagtggtttaaactatcag

tgtttgacaggatatattggcgggtaaacctaagagaaaagagcgtttattagaataatcggatatttaaaagggcgtga

aaaggtttatccgttcgtccatttgtatgtgcatgccaaccacagggttccccagatcaggcgctggctgctgaaccccc

agccggaactgaccccacaaggccctagcgtttgcaatgcaccaggtcatcattgacccaggcgtgttccaccaggccgc

tgcctcgcaactcttcgcaggcttcgccgacctgctcgcgccacttcttcacgcgggtggaatccgatccgcacatgagg

cggaaggtttccagcttgagcgggtacggctcccggtgcgagctgaaatagtcgaacatccgtcgggccgtcggcgacag

cttgcggtacttctcccatatgaatttcgtgtagtggtcgccagcaaacagcacgacgatttcctcgtcgatcaggacct

ggcaacgggacgttttcttgccacggtccaggacgcggaagcggtgcagcagcgacaccgattccaggtgcccaacgcgg

tcggacgtgaagcccatcgccgtcgcctgtaggcgcgacaggcattcctcggccttcgtgtaataccggccattgatcga

ccagcccaggtcctggcaaagctcgtagaacgtgaaggtgatcggctcgccgataggggtgcgcttcgcgtactccaaca

cctgctgccacaccagttcgtcatcgtcggcccgcagctcgacgccggtgtaggtgatcttcacgtccttgttgacgtgg

aaaatgaccttgttttgcagcgcctcgcgcgggattttcttgttgcgcgtggtgaacagggcagagcgggccgtgtcgtt

tggcatcgctcgcatcgtgtccggccacggcgcaatatcgaacaaggaaagctgcatttccttgatctgctgcttcgtgt

gtttcagcaacgcggcctgcttggcctcgctgacctgttttgccaggtcctcgccggcggtttttcgcttcttggtcgtc

atagttcctcgcgtgtcgatggtcatcgacttcgccaaacctgccgcctcctgttcaagacgacgcgaacgctccacggc

ggccgatggcgcgggcagggcagggggagccagttgcacgctgtcgcgctcgatcttggccgtagcttgctggaccatcg

agccgacggactggaaggtttcgcggggcgcacgcatgacggtgcggcttgcgatggtttcggcatcctcggcggaaaac

cccgcgtcgatcagttcttgcctgtatgccttccggtcaaacgtccgattcattcaccctccttgcgggattgccccgac

tcacgccggggcaatgtgcccttattcctgatttgacccgcctggtgccttggtgtccagataatccaccttatcggcaa

tgaagtcggtcccgtagaccgtctggccgtccttctcgtacttggtattccgaatcttgccctgcacgaataccagcgac

cccttgcccaaatacttgccgtgggcctcggcctgagagccaaaacacttgatgcggaagaagtcggtgcgctcctgctt

gtcgccggcatcgttgcgccacatctaggatctgccaggaaccgtaaaaaggccgcgttgctggcgtttttccataggct

ccgcccccctgacgagcatcacaaaaatcgacgctcaagtcagaggtggcgaaacccgacaggactataaagataccagg

cgtttccccctggaagctccctcgtgcgctctcctgttccgaccctgccgcttaccggatacctgtccgcctttctccct

tcgggaagcgtggcgctttctcatagctcacgctgtaggtatctcagttcggtgtaggtcgttcgctccaagctgggctg

tgtgcacgaaccccccgttcagcccgaccgctgcgccttatccggtaactatcgtcttgagtccaacccggtaagacacg

acttatcgccactggcagcagccactggtaacaggattagcagagcgaggtatgtaggcggtgctacagagttcttgaag

tggtggcctaactacggctacactagaaggacagtatttggtatctgcgctctgctgaagccagttaccttcggaaaaag

agttggtagctcttgatccggcaaacaaaccaccgctggtagcggtggtttttttgtttgcaagcagcagattacgcgca

gaaaaaaaggatctcaagaagatcctttgatcttttctacggggtctgacgctcagtggaacgaaaactcacgttaaggg

attttggtcatgagattatcaaaaaggatcttcacctagatccttttaaattaaaaatgaagttttaaatcaatctaaag

tatatatgagtaaacttggtctgacagttaccaatgcttaatcagtgaggcacctatctcagcgatctgtctatttcgtt

catccatagttgcctgactccccgtcgtgtagataactacgatacgggagggcttaccatctggccccagtgctgcaatg

ataccgcgagaaccacgctcaccggctccagatttatcagcaataaaccagccagccggaagggccgagcgcagaagtgg

tcctgcaactttatccgcctccatccagtctattaattgttgccgggaagctagagtaagtagttcgccagttaatagtt

tgcgcaacgttgttgccattgctacaggcatcgtggtgtcacgctcgtcgtttggtatggcttcattcagctccggttcc

caacgatcaaggcgagttacatgatcccccatgttgtgcaaaaaagcggttagctccttcggtcctccgatcgttgtcag

aagtaagttggccgcagtgttatcactcatggttatggcagcactgcataattctcttactgtcatgccatccgtaagat

gcttttctgtgactggtgagtactcaaccaagtcattctgagaatagtgtatgcggcgaccgagttgctcttgcccggcg

tcaatacgggataataccgcgccacatagcagaactttaaaagtgctcatcattggaaaacgttcttcggggcgaaaact

ctcaaggatcttaccgctgttgagatccagttcgatgtaacccactcgtgcacccaactgatcttcagcatcttttactt

tcaccagcgtttctgggtgagcaaaaacaggaaggcaaaatgccgcaaaaaagggaataagggcgacacggaaatgttga

atactcatactcttcctttttcaatattattgaagcatttatcagggttattgtctcatgagcggatacatatttgaatg

tatttagaaaaataaacaaataggggttccgcgcacgaattggccagcgctgccatttttggggtgaggccgttcgcggc

cgaggggcgcagcccctggggggatgggaggcccgcgttagcgggccgggagggttcgagaagggggggcaccccccttc

ggcgtgcgcggtcacgcgcacagggcgcagccctggttaaaaacaaggtttataaatattggtttaaaagcaggttaaaa

gacaggttagcggtggccgaaaaacgggcggaaacccttgcaaatgctggattttctgcctgtggacagcccctcaaatg

tcaataggtgcgcccctcatctgtcagcactctgcccctcaagtgtcaaggatcgcgcccctcatctgtcagtagtcgcg

cccctcaagtgtcaataccgcagggcacttatccccaggcttgtccacatcatctgtgggaaactcgcgtaaaatcaggc

gttttcgccgatttgcgaggctggccagctccacgtcgccggccgaaatcgagcctgcccctcatctgtcaacgccgcgc

cgggtgagtcggcccctcaagtgtcaacgtccgcccctcatctgtcagtgagggccaagttttccgcgaggtatccacaa

cgccggcggccgcggtgtctcgcacacggcttcgacggcgtttctggcgcgtttgcagggccatagacggccgccagccc

agcggcgagggcaaccagcccggtgagcgtcgcaaaggagatcctgatctgactgatgggctgcctgtatcgagtggtga

ttttgtgccgagctgccggtcggggagctgttggctggctggtggcaggatatattgtggtgtaaacaaattgacgctta

gacaacttaataacacattgcggacgtttttaatgtactggggtggatgcagtgggccccac

Features :

RK2\trfa\(no\Esp3I) : [3223 : 1742 - CCW]

RB\short : [1718 : 1591 - CCW]

shows similarity to T-DNA left border: GenBank Accession Number J01825_TDNA-LB : [5733 : 5880 - CW]

shows similarity to GenBank Accession Number M20134_oriV : [5703 : 5086 - CCW]

pUC\ori : [3234 : 4023 - CW]

RNaseH cleavage point_ORI : [3269 : 3269 - CW]

AP\r : [4888 : 4031 - CCW]

RbcS2p_regulatory : [307 : 527 - CW]

CrNPTII : [529 : 1320 - CW]

tRbcS2 : [1325 : 1558 - CW]

pHsp70A : [34 : 300 - CW]

5UTR CrRbcS2 : [505 : 527 - CW]

pRbcS2 : [312 : 504 - CW]

ColE1 origin : [3879 : 3251 - CCW]

Amp prom : [4958 : 4930 - CCW]

BbsI : [6 : 11 - CW]

BbsI : [1575 : 1570 - CCW]
