# Supplementary material for: Blasticidin S Deaminase: A New Efficient Selectable Marker for Chlamydomonas reinhardtii
Source: Front Plant Sci. 2020 Mar 5;11:242. doi: 10.3389/fpls.2020.00242 (PMC7066984; doi:10.3389/fpls.2020.00242)
Supplement: FILE S7 — Annotated sequence of pCM1-029, the level 1 plasmid made up of the promoter AR (PA/R + 5′UTR of RBCS2), aadA coding sequence and the terminator RBCS2 (3′UTR of RBCS2 + TRBCS2) conferring spectinomycin resistance in Chlamydomonas (Meslet-Cladière and Vallon, 2011; Crozet et al., 2018). [file Data_Sheet_7.docx]

> [pCM1-033-pAR-aadA-tRbcS2.xdna - 6136 bp] Ligation of : pL1-1F (pICH47732).xdna [4368 nt] : (#BsaI[619] / #BsaI[35]) to i1-28.xdna [1760 nt] : (Uncut 5'[0] / Uncut 3'[1760])

tctgtgaagacaatgccgaattcggatccggaggctgaggcttgacatgattggtgcgtatgtttgtatgaagctacagg

actgatttggcgggctatgagggcgggggaagctctggaagggccgcgatggggcgcgcggcgtccagaaggcgccatac

ggcccgctggcggcacccatccggtataaaagcccgcgaccccgaacggtgacctccactttcagcgacaaacgagcact

tatacatacgcgactattctgccgctatacataaccactcagctagcttaagatcccatcaagcttgcatgccgggcgcg

ccagaaggagcgcagccaaaccaggatgatgtttgatggggtatttgagcacttgcaacccttatccggaagccccctgg

cccacaaaggctaggcgccaatgcaagcagttcgcatgcagcccctggagcggtgccctcctgataaaccggccaggggg

cctatgttctttacttttttacaagagaagtcactcaacatcttaaaaatgGCCATGGCGGCCGTGATCGCCAAGTCGAG

CGTGAGCGCCGCCGTGGCCCGCCCCGCCCGCTCCAGCGTGCGCCCCATGGCCGCCTTGAAGCCCGCCGTGAAGGCCGCGC

CCGTGGCCGCGCCCGCCCAAGCCAACCAGCAATTGATGGCCATGCGCACCCCGGAGGAGCTGTCCAACCTGATTAAGGAT

CTGATCGAGCAGTACACTCCCGAGGTCAAGATGTCGATGGCTCGGGAGGCCGTGATTGCGGAGGgCTCGACCCAGCTGAG

CGAAGTCGTGGGCGTCATCGAGCGCCACCTGGAGCCCACCCTGCTGGCCGTGCACCTGTACGGCTCCGCCGTGGACGGGG

GCCTGAAGCCCCACTCGGACATCGACCTGCTCGTGACCGTGACCGTGCGCCTGGACGAGACTACTCGCCGGGCTCTCATC

AACGACCTGCTGGAAACGAGCGCGTCGCCTGGCGAGTCGGAGATCCTGCGCGCCGTGGAAGTCACCATCGTCGTGCATGA

CGACATTATCCCCTGGCGCTACCCGGCCAAGCGCGAGCTGCAATTCGGCGAGTGGCAGCGCAACGACATCCTGGCCGGCA

TCTTCGAGCCCGCGACCATTGACATCGACCTGGCGATCCTCCTGACGAAGGCCCGCGAGCACTCCGTGGCGCTCGTCGGC

CCGGCGGCGGAGGAGCTGTTTGACCCCGTGCCGGAGCAGGACCTGTTCGAGGCTCTGAACGAaACCCTGACGCTGTGGAA

CTCCCCTCCGGATTGGGCCGGCGACGAGCGGAACGTCGTGCTGACCCTGAGCCGCATTTGGTATTCGGCGGTCACCGGCA

AGATCGCCCCCAAGGACGTGGCGGCGGACTGGGCCATGGAGCGGCTGCCGGCGCAATACCAGCCCGTGATCCTGGAGGCC

CGGCAAGCCTACCTCGGGCAGGAGGAGGACCGCCTGGCGAGCCGGGCGGACCAGCTGGAGGAGTTCGTGCACTACGTCAA

GGGCGAGATCACGAAGGTCGTGGGCAGTATCTAGgcttccgctccgtgtaaatggAGGCGCTCGTTGATCTGAGCCTTGC

CCCCTGACGAACGGCGGTGGATGGAAGATACTGCTCTCAAGTGCTGAAGCGGTAGCTTAGCTCCCCGTTTCGTGCTGATC

AGTCTTTTTCAACACGTAAAAAGCGGAGGAGTTTTGCAATTTTGTTGGTTGTAACGATCCTCCGTTGATTTTGGCCTCTT

TCTCCATGGGCGGGCTgggcgtatttgaagcggcgctgcaattgtcttctgcacgaagtggtttaaactatcagtgtttg

acaggatatattggcgggtaaacctaagagaaaagagcgtttattagaataatcggatatttaaaagggcgtgaaaaggt

ttatccgttcgtccatttgtatgtgcatgccaaccacagggttccccagatcaggcgctggctgctgaacccccagccgg

aactgaccccacaaggccctagcgtttgcaatgcaccaggtcatcattgacccaggcgtgttccaccaggccgctgcctc

gcaactcttcgcaggcttcgccgacctgctcgcgccacttcttcacgcgggtggaatccgatccgcacatgaggcggaag

gtttccagcttgagcgggtacggctcccggtgcgagctgaaatagtcgaacatccgtcgggccgtcggcgacagcttgcg

gtacttctcccatatgaatttcgtgtagtggtcgccagcaaacagcacgacgatttcctcgtcgatcaggacctggcaac

gggacgttttcttgccacggtccaggacgcggaagcggtgcagcagcgacaccgattccaggtgcccaacgcggtcggac

gtgaagcccatcgccgtcgcctgtaggcgcgacaggcattcctcggccttcgtgtaataccggccattgatcgaccagcc

caggtcctggcaaagctcgtagaacgtgaaggtgatcggctcgccgataggggtgcgcttcgcgtactccaacacctgct

gccacaccagttcgtcatcgtcggcccgcagctcgacgccggtgtaggtgatcttcacgtccttgttgacgtggaaaatg

accttgttttgcagcgcctcgcgcgggattttcttgttgcgcgtggtgaacagggcagagcgggccgtgtcgtttggcat

cgctcgcatcgtgtccggccacggcgcaatatcgaacaaggaaagctgcatttccttgatctgctgcttcgtgtgtttca

gcaacgcggcctgcttggcctcgctgacctgttttgccaggtcctcgccggcggtttttcgcttcttggtcgtcatagtt

cctcgcgtgtcgatggtcatcgacttcgccaaacctgccgcctcctgttcaagacgacgcgaacgctccacggcggccga

tggcgcgggcagggcagggggagccagttgcacgctgtcgcgctcgatcttggccgtagcttgctggaccatcgagccga

cggactggaaggtttcgcggggcgcacgcatgacggtgcggcttgcgatggtttcggcatcctcggcggaaaaccccgcg

tcgatcagttcttgcctgtatgccttccggtcaaacgtccgattcattcaccctccttgcgggattgccccgactcacgc

cggggcaatgtgcccttattcctgatttgacccgcctggtgccttggtgtccagataatccaccttatcggcaatgaagt

cggtcccgtagaccgtctggccgtccttctcgtacttggtattccgaatcttgccctgcacgaataccagcgaccccttg

cccaaatacttgccgtgggcctcggcctgagagccaaaacacttgatgcggaagaagtcggtgcgctcctgcttgtcgcc

ggcatcgttgcgccacatctaggatctgccaggaaccgtaaaaaggccgcgttgctggcgtttttccataggctccgccc

ccctgacgagcatcacaaaaatcgacgctcaagtcagaggtggcgaaacccgacaggactataaagataccaggcgtttc

cccctggaagctccctcgtgcgctctcctgttccgaccctgccgcttaccggatacctgtccgcctttctcccttcggga

agcgtggcgctttctcatagctcacgctgtaggtatctcagttcggtgtaggtcgttcgctccaagctgggctgtgtgca

cgaaccccccgttcagcccgaccgctgcgccttatccggtaactatcgtcttgagtccaacccggtaagacacgacttat

cgccactggcagcagccactggtaacaggattagcagagcgaggtatgtaggcggtgctacagagttcttgaagtggtgg

cctaactacggctacactagaaggacagtatttggtatctgcgctctgctgaagccagttaccttcggaaaaagagttgg

tagctcttgatccggcaaacaaaccaccgctggtagcggtggtttttttgtttgcaagcagcagattacgcgcagaaaaa

aaggatctcaagaagatcctttgatcttttctacggggtctgacgctcagtggaacgaaaactcacgttaagggattttg

gtcatgagattatcaaaaaggatcttcacctagatccttttaaattaaaaatgaagttttaaatcaatctaaagtatata

tgagtaaacttggtctgacagttaccaatgcttaatcagtgaggcacctatctcagcgatctgtctatttcgttcatcca

tagttgcctgactccccgtcgtgtagataactacgatacgggagggcttaccatctggccccagtgctgcaatgataccg

cgagaaccacgctcaccggctccagatttatcagcaataaaccagccagccggaagggccgagcgcagaagtggtcctgc

aactttatccgcctccatccagtctattaattgttgccgggaagctagagtaagtagttcgccagttaatagtttgcgca

acgttgttgccattgctacaggcatcgtggtgtcacgctcgtcgtttggtatggcttcattcagctccggttcccaacga

tcaaggcgagttacatgatcccccatgttgtgcaaaaaagcggttagctccttcggtcctccgatcgttgtcagaagtaa

gttggccgcagtgttatcactcatggttatggcagcactgcataattctcttactgtcatgccatccgtaagatgctttt

ctgtgactggtgagtactcaaccaagtcattctgagaatagtgtatgcggcgaccgagttgctcttgcccggcgtcaata

cgggataataccgcgccacatagcagaactttaaaagtgctcatcattggaaaacgttcttcggggcgaaaactctcaag

gatcttaccgctgttgagatccagttcgatgtaacccactcgtgcacccaactgatcttcagcatcttttactttcacca

gcgtttctgggtgagcaaaaacaggaaggcaaaatgccgcaaaaaagggaataagggcgacacggaaatgttgaatactc

atactcttcctttttcaatattattgaagcatttatcagggttattgtctcatgagcggatacatatttgaatgtattta

gaaaaataaacaaataggggttccgcgcacgaattggccagcgctgccatttttggggtgaggccgttcgcggccgaggg

gcgcagcccctggggggatgggaggcccgcgttagcgggccgggagggttcgagaagggggggcaccccccttcggcgtg

cgcggtcacgcgcacagggcgcagccctggttaaaaacaaggtttataaatattggtttaaaagcaggttaaaagacagg

ttagcggtggccgaaaaacgggcggaaacccttgcaaatgctggattttctgcctgtggacagcccctcaaatgtcaata

ggtgcgcccctcatctgtcagcactctgcccctcaagtgtcaaggatcgcgcccctcatctgtcagtagtcgcgcccctc

aagtgtcaataccgcagggcacttatccccaggcttgtccacatcatctgtgggaaactcgcgtaaaatcaggcgttttc

gccgatttgcgaggctggccagctccacgtcgccggccgaaatcgagcctgcccctcatctgtcaacgccgcgccgggtg

agtcggcccctcaagtgtcaacgtccgcccctcatctgtcagtgagggccaagttttccgcgaggtatccacaacgccgg

cggccgcggtgtctcgcacacggcttcgacggcgtttctggcgcgtttgcagggccatagacggccgccagcccagcggc

gagggcaaccagcccggtgagcgtcgcaaaggagatcctgatctgactgatgggctgcctgtatcgagtggtgattttgt

gccgagctgccggtcggggagctgttggctggctggtggcaggatatattgtggtgtaaacaaattgacgcttagacaac

ttaataacacattgcggacgtttttaatgtactggggtggatgcagtgggccccac

Features :

RK2\trfa\(no\Esp3I) : [3457 : 1976 - CCW]

RB\short : [1952 : 1825 - CCW]

shows similarity to T-DNA left border: GenBank Accession Number J01825_TDNA-LB : [5967 : 6114 - CW]

shows similarity to GenBank Accession Number M20134_oriV : [5937 : 5320 - CCW]

pUC\ori : [3468 : 4257 - CW]

RNaseH cleavage point_ORI : [3503 : 3503 - CW]

AP\r : [5122 : 4265 - CCW]

pHsp70 : [34 : 301 - CW]

RbcS2p_regulatory : [307 : 527 - CW]

AadA-SpecR : [532 : 1554 - CW]

TRbcS2 : [1559 : 1793 - CW]

5UTR CrRBCS2 : [505 : 527 - CW]

pRbcS2 : [312 : 504 - CW]

ColE1 origin : [4113 : 3485 - CCW]

Amp prom : [5192 : 5164 - CCW]

BbsI : [6 : 11 - CW]

BbsI : [1809 : 1804 - CCW]
